# Rejuvenating bone marrow hematopoietic reserve prevents regeneration failure and hepatic decompensation in animal model of cirrhosis

**DOI:** 10.3389/fimmu.2024.1439510

**Published:** 2024-08-12

**Authors:** Nidhi Nautiyal, Deepanshu Maheshwari, Dhananjay Kumar, E. Pranshu Rao, Dinesh Mani Tripathi, Sandeep Kumar, Sunidhi Diwakar, Manisha Bhardwaj, Sujata Mohanty, Prakash Baligar, Anupama Kumari, Chhagan Bihari, Subhrajit Biswas, S. K. Sarin, Anupam Kumar

**Affiliations:** ^1^ Department of Molecular and Cellular Medicine, Institute of Liver and Biliary Sciences, New Delhi, India; ^2^ Amity Institute of Molecular Medicine and Stem Cell Research, Amity University, Noida, India; ^3^ Stem Cell Facility, All India Institute of Medical Sciences, New Delhi, India; ^4^ Department of Pathology, Institute of Liver and Biliary Sciences, New Delhi, India; ^5^ Department of Hepatology, Institute of Liver and Biliary Sciences, New Delhi, India

**Keywords:** chronic liver injury, regeneration failure, hematopoietic stem cells, hepatic decompensation, kupffer cells, cirrhosis, innate immune response

## Abstract

**Background and aim:**

Bone marrow stem cells (BM-SCs) and their progeny play a central role in tissue repair and regeneration. In patients with chronic liver failure, bone marrow (BM) reserve is severally compromised and they showed marked defects in the resolution of injury and infection, leading to liver failure and the onset of decompensation. Whether BM failure is the cause or consequence of liver failure during cirrhosis is not known. In this study, we aimed to determine the underlying relationship between BM failure and regeneration failure in cirrhosis.

**Methodology:**

C57Bl/6(J) mice were used to develop chronic liver injury through intra-peritoneal administration of carbon tetrachloride (CCl4) for 15 weeks (0.1-0.5 ml/kg). Animals were sacrificed to study the transition of cirrhosis and BM defects. To restore the BM-SC reserve; healthy BM cells were infused via intra-BM infusion and assessed for changes in liver injury, regeneration, and BM-SC reserve.

**Results:**

Using a CCl4-induced animal - model of cirrhosis, we showed the loss of BM-SCs reserve occurred before regeneration failure and the onset of non-acute decompensation. Intra-BM infusion of healthy BM cells induced the repopulation of native hematopoietic stem cells (HSCs) in cirrhotic BM. Restoring BM-HSCs reserve augments liver macrophage-mediated clearance of infection and inflammation dampens neutrophil-mediated inflammation, accelerates fibrosis regression, enhances hepatocyte proliferation, and delays the onset of non-acute decompensation.

**Conclusion:**

These findings suggest that loss of BM-HSCs reserve underlies the compromised innate immune function of the liver, drives regeneration failure, and the onset of non-acute decompensation. We further provide the proof-of-concept that rejuvenating BM-HSC reserve can serve as a potential therapeutic approach for preventing regeneration failure and transition to decompensated cirrhosis.

## Introduction

Chronic liver injury is a slow progressive disease that often progresses to cirrhosis and slowly leads to portal hypertension (PH) and hepatic decompensation (1–3). The underlying mechanisms of the transition from compensated-to-decompensated cirrhosis are an area of active investigation. Emerging evidence on the pathophysiology of cirrhosis highlights the role of portal hypertension (4), exacerbated systemic inflammation (5, 6), increased intestinal bacterial translocation (7–11) and regeneration failure (12–14) in this transition. The mechanisms underlying this poor resolution of liver injury/infection and regeneration failure during cirrhosis are not well defined.

Demand-adapted hematopoiesis, particularly increased production of myeloid cells, plays a central role in meeting the increased demand of myeloid cells, required for the effective clearance of liver injury/infection, and orchestrates the niche for effective liver regeneration (9, 15–19). Despite increased myelopoiesis, why innate immune-mediated clearance of injury/infection is compromised in cirrhosis is not clearly defined. Emerging data over recent years suggested the potential role of “innate immune memory” that leads to either exaggerated (trained immunity) or impaired (tolerance) innate immune cell function in chronic injury (20–23). Although not well defined, but some lines of evidence suggested the potential link between innate immune memory and impairment of monocyte/macrophage dysfunction in cirrhosis (24).While trained immunity might have role in driving initial inflammation-based disease progression (25), in later stages immune tolerance (24)/paralysis (26) may lead to increased innate immune dysfunction. Patients with liver cirrhosis exhibit broad defects in bone marrow stem cells (BM-SCs) (27–30), and their progeny (31), required for the adequate clearance of injury and infection. Therapeutic use of BM hematopoietic stem cells (HSC) has been shown to improve the regression of fibrosis and regeneration and transiently improves the outcome of cirrhotic patients with preserved HSC reserves (32). Together these facts highlight the association between defective BM-SC reserves and poor resolution of injury and regeneration associated with cirrhosis. The underlying relationship between defects in BM-SC reserves, liver repair, and regeneration failure during chronic liver injury is unknown.

To address whether the loss of the BM-SC reserve is the cause or consequence of liver failure, we examined the kinetic change in BM hematopoiesis and hepatic regeneration during the course of chronic liver injury. Given the crucial role of BM in tissue repair and regeneration, we also evaluated the therapeutic benefits of restoring the BM-HSCs reserve to promote the resolution of liver injury and regeneration during cirrhosis.

## Materials and methods

### Ethics statement

The animal studies were conducted in accordance with the guidelines by the Committee for the Purpose of Control and Supervision of Experiments on Animals (CPCSEA) Govt. of India and were approved by the Institutional Animal Ethics Committee (IAEC), Institute of Liver and Biliary Sciences, New Delhi, India (IAEC/ILBS/17/02). All investigations were conducted following the ARRIVE (Animal Research: Reporting of *In Vivo* Experiments) protocols and the principles outlined in the Basel Declaration (http://www.basel.declaration.org), which encompass the 3R principle.

### Disease model

Wild-type C57Bl/6 mice (male, 6–8 weeks) were purchased from an institutional animal facility and housed in a clean, temperature-controlled environment with a 12H light and dark cycle, provided with free access to a regular laboratory chow diet and water. The Institutional Animal Ethics Committee (IAEC; IAEC/ILBS/17/02) approved all animal care and experimental procedures. Chronic liver injury induced by intraperitoneal (i.p.) injections of carbon tetrachloride (CCL4, Central Drug House, Delhi, India) in olive oil (HiMedia Pvt. Ltd., India) started at a dose of (0.1–0.5) ml/kg of body weight and were given twice weekly for 15 weeks. Mice were sacrificed post week-3/6/10/15 to know the fibrosis level during chronic liver injury and compared with the controls (received i.p. olive oil). The animals were studied for the progression of hepatic decompensation.

### Cell therapy

After completing the 11th week of chronic CCl4 treatment, mice were divided into different groups based on the experimental design. Set 1: To study the mode of cell therapy, mice were divided into three groups: group-1 received IF-BM infusion, group-2 received intravenous (IV) BM infusion, and group-3 served as the vehicle control (received incomplete RPMI media); mice were sacrificed at 24H, D11 and D21 of post-cells infusion. Set 2: To study the effectiveness of cell therapy in restoring native BM-HSC reserve and impact on liver repair and regeneration. Group-1 received cells from syngeneic healthy C57Bl/6-GFP, Group-2 received IF-BM cells from syngeneic cirrhotic (10-weeks of CCl4 injury) C57Bl/6-GFP while group-3 was vehicle control (received incomplete RPMI media). Set 3: To study the infused BM cells’ ability to ameliorate the progression of decompensated cirrhosis. Cells were isolated from Femurs and tibias and labelled with DIR (#D12731) dye for ex-vivo imaging. Approximately, 4X106 cells were infused per mice intra-femorally.

### Method of euthanasia

At the time of sacrifice, mice were deeply anaesthetized with the cocktail of ketamine (50mg/ml), xylazine (20mg/ml) and saline (0.9% V/V) by i.p injection (0.1ml/20gm of mice).

### Cells preparation

Femurs and tibias were removed from 6-8 weeks aged healthy or cirrhotic C57BL/6-Tg (UBC-GFP)30Scha/J. BM cells were extracted from both the bones and a single cell suspension was prepared by passing through a 75µm filter. The cells were further labelled with DIR (#D12731) dye for ex-vivo imaging. Approximately, 4X106 cells were infused per mice.

### Ex-vivo imaging

DIR, 1, 1-dioctadecyl-3,3,3,3-tetramethylindotricarbocyanine iodide (#D12731, Invitrogen, Life Technologies, USA), used to tag cells at 10µM concentration for 15minutes at room temperature and washed with 1XPBS. These GFP+ DIR tagged cells were used to see the migration of donor’s cells. At every time point (24H, D11 and D21) mice were euthanized. Imaging was done using AIIMS IVIS facility, New Delhi, India.

### Blood serum biochemistry

Blood was collected via retro-orbital puncture, in a blood collecting tube and centrifuged for 15minutes at 3000 rpm to collect serum. The serum levels for liver and kidney injury were determined by the biochemical analyzer at Institutional facility.

### Flow cytometry analysis

Cells were analyzed based on surface markers: LSK (LIN-/c-Kit+/SCA-1+; HSPCs); LT-HSC (LIN-/c-Kit+/SCA-1+/FLT3-/CD34-); ST-HSC (LIN-/c-Kit+/SCA-1+/FLT3-/CD34+); and MPPs (LIN-/c-Kit+/SCA-1+/FLT+/CD34+) for HSCs. For MSCs: TER119-/CD45-/CD31-/NESTIN+. Liver kupffer cells were analyzed based on F4/80+ surface marker and checked for phagocytosis (Cayman Phagocytosis assay kit as per the manufacturer protocol).

### Cells staining

Cells were incubated with an antibody cocktail for 40 minutes at 4°C, washed with 1XPBS and acquired using FACS Verse, and analyzed using FlowJo (Treestar Inc., V10) for the FCS file.

### Colony forming unit-fibroblasts

The CFU-F assay involved culturing 2X10^6 bone marrow cells per well in a six-well plate in triplicates. On Day 11, the cells were washed, fixed (4% paraformaldehyde, 20 minutes), and stained with 0.5% crystal violet for 15-20 minutes before being counted.

### Hemodynamic assessment

Animals were anesthetized with isoflurane inhalation for assessment of portal pressure; the ileocolic vein was cannulated with PE-10 catheters connected to a pressure transducer (Edwards Life Sciences, Irvine, CA), and the pressure transducers were connected to a PowerLab (4SP) linked to a computer using the Chart version 5.0.1 for Windows software (AD Instruments, Australia). The temperature of the animals was maintained at 37 ± 0.5°C. Hemodynamic data were collected after 8–10 minutes of the stabilization period.

### Histopathology and immunohistochemistry

Liver and femur (decalcified using 14% EDTA at 4oC for 3-4 days) tissues were fixed, processed using paraffin block techniques, and stained with H&E, Sirius red, and MT. IHC was performed to study liver regeneration and fibrosis. Paraffin-embedded sections were stained with primary antibodies (PCNA, Collagen I, α-SMA, F4/80, Nestin+). DAB substrate and streptavidin-horseradish peroxidase were used for visualization. TUNEL assay was performed using *in-situ* Cell Death Detection Kit (Roche #11684795910) as per the manufacturer’s protocol.

### Endotoxin assay

The “Pierce Chromogenic Endotoxin Quant Kit” (ThermoFisher#A39553) is used as per the manufacturer’s protocol to analyze the endotoxin level in mouse samples.

### Efferocytosis

GFP+ Neutrophils from GFP+ mice isolated through gradient centrifugation (via GranuloSep and HiSep, 700g 30mins) from blood. Apoptosis was induced by UV exposure for 10mins in neutrophils while incubated for 45mins in RPMI complete media (10%), wash twice with 1X PBS. The ratio was maintained at Macrophages: Apoptotic cells (M:AC; 1:3 respectively) and acquired through flow cytometer and images were taken through confocal microscope.

### Quantitative reverse transcriptase-PCR

The total RNA was extracted from snap-frozen liver tissue using TRIzol (Invitrogen, Shanghai, China) method as per the protocol. RNA concentrations were measured with NanoDrop ND-100 spectrophotometer (NanoDrop Technologies Thermo Scientific). Polymerase chain reaction (PCR) was performed using 2 µg of RNA to generate cDNA (ABI, Invitrogen) which was used as the template, and combined with standard SYBR Green (Kappa) on the Real-Time PCR Detection System (Biorad). All reactions were performed in triplicate and the data were analyzed using the 2-ΔΔCt method.

### Mass spectrometry

#### Sample preparation

Proteins were isolated from liver tissue (approximately 150mg) and BM sorted LSK cells (approximately 1 x 10^5) using RIPA buffer (with Proteinase K). Proteins were estimated by Bradford’s method. 50µg of protein sample was used for digestion and reduced with 5mM TCEP and further alkylated with 50mM iodoacetamide and then digested with Trypsin (1:50, Trypsin/lysate ratio) for 16h at 37°C. Digests were cleaned using a C18 silica cartridge to remove the salt and dried using a speed vac. The dried pellet was resuspended in bufferA (2% acetonitrile, 0.1% formic acid).

#### Mass spectrometric analysis of peptide mixtures

All the experiments were performed using EASY-nLC 1200 system (Thermo Fisher Scientific) coupled to Thermo Fisher-QExactive plus equipped with nano electrospray ion source. 1µg was loaded on C18 column 50cm, 3.0μm Easy-spray column (ThermoFisher Scientific). Peptides were eluted with a 0–40% gradient of bufferB (80% acetonitrile, 0.1% formic acid) at a flow rate of 300 nl/min) and injected for MS analysis. LC gradients were run for 60 minutes. MS1 spectra were acquired in the Orbitrap at 70k resolution. Dynamic exclusion was employed for 10 s excluding all charge states for a given precursor. MS2 spectra were acquired at 17500 resolutions. (Protein isolation and proteomics run/analysis was done by Valerian Chem Private Limited, New Delhi, Delhi 110066, India).

#### Data processing

All the samples once processed, were subjected to mass spectrometry run. Raw files containing mass/charge values were generated for each of the samples. Further these raw files were analyzed through Thermo Proteome Discoverer (v2.2) against mouse proteome database. For Sequest and Amanda (ML algorithms used to map peptides identified by mass spectrometer against the protein database) search, the precursor and fragment mass tolerance were set at 10 ppm and 0.02 Da, respectively. The protease used to generate peptides, i.e. enzyme specificity was set for trypsin/P (cleavage at the C terminus of “K/R: unless followed by “P”) along with maximum missed cleavages value of two. Carbamidomethyl on cysteine as fixed modification and oxidation of methionine were considered as Variable modifications for database search. Both peptide spectrum match and protein false discovery rate were set to 0.01 FDR. PD output is generally in the form of a matrix that contains accession IDs and abundance values for each of the identified proteins per sample, along with relevant annotation for each identified protein. Peptides and proteins sheet are exported individually and then are used for downstream analysis.

### Statistical analysis

Differential Expression Analysis: Raw abundance values and accession IDs are extracted from within the matrix. This matrix is used for differential expression analysis for the identification of significant proteins. For Differential Expression Analysis between different groups for liver and BM samples, the data was first transformed on log2 scale and filtered based on valid values (features which were quantified in 70% of the samples). The remaining values after filtering were imputed based on the normal distribution and then normalized using MBQN. Once the data is normalized, t-test analysis is performed that provides pValues and logFC (log2 Fold Change) values. The proteins that have pvalues<0.05 are considered significant. Volcano plots were generated using log10 pValues and log2 Fold change values.

All sections (liver, lungs, kidney and spleen were evaluated in a blind manner by the pathologist and were examined using EVOS@FL light microscopy. Micrographs quantified using ImageJ (FJI, 1.47V) in three non-overlapping random fields and dot plots were made using R-studio (RStudio, PBC, 4.2.1). Data are presented as mean ± standard error of the mean. Two-tailed Student’s t and Mann-Whitney U tests were used to analyze parametric and nonparametric data, respectively using Prism (Graph-Pad Software, Inc, 6.01) unless otherwise stated.

## Results

### The loss of BM-HSCs precedes regeneration failure and the onset of decompensated cirrhosis

**Figure 1:**
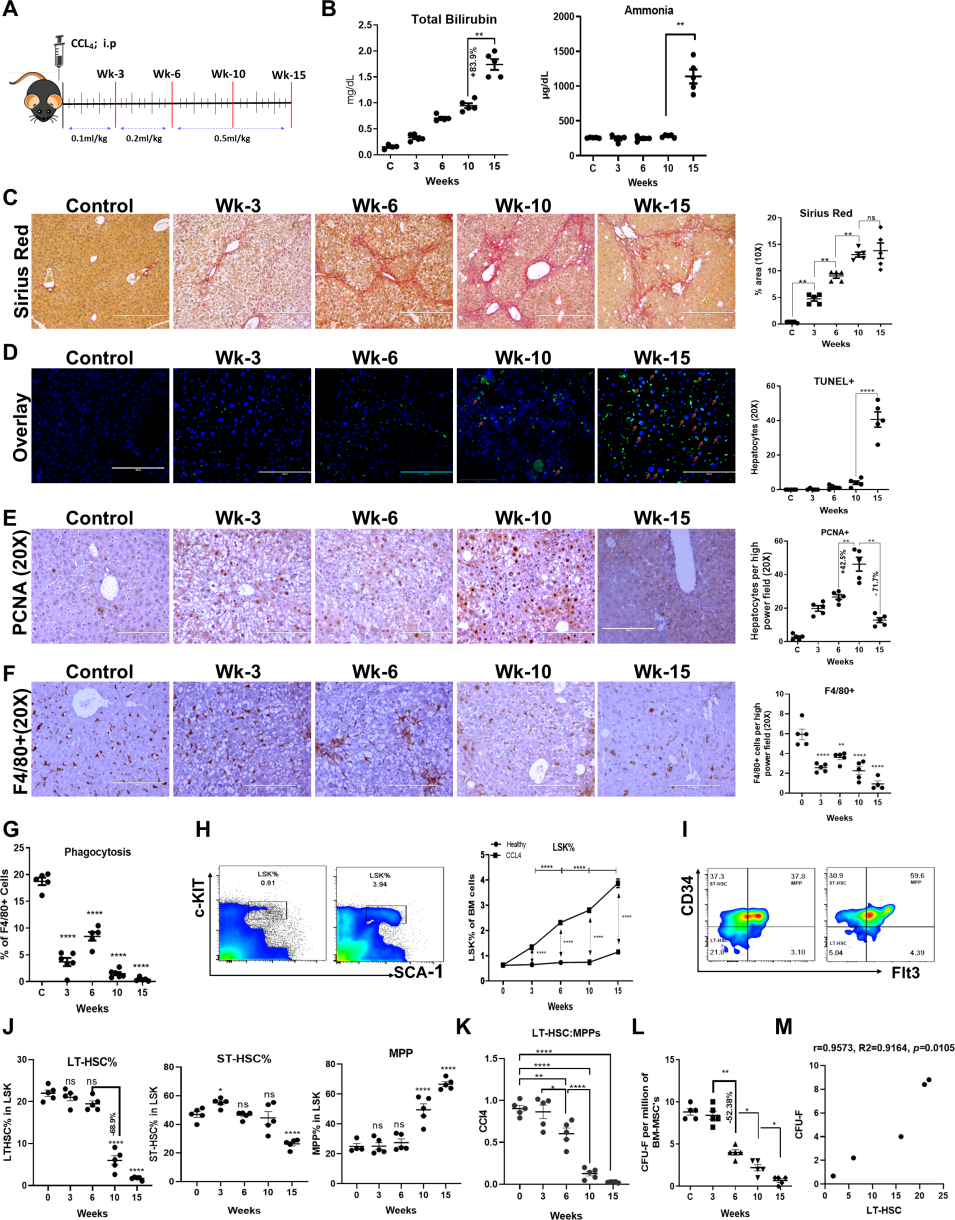
Loss of BM-HSC reserve precedes regeneration failure and the onset of decompensated cirrhosis. **(A)** Schematic representation of a progressive mouse model of chronic liver injury. **(B)** The blood biochemical levels of total bilirubin and ammonia at each time point, (N = 5). **(C)** The liver sections were stained with Sirius red and quantitate for progressive change in fibrosis between the groups (N = 5). **(D)** The Representative immunofluorescent staining images of TUNEL+ hepatocytes (GFP+) and its quantitative analysis (20X) (N = 5). **(E)** The micrographs showing the immunohistochemistry for hepatocyte proliferation (PCNA staining; 20X) at every stage of CCL4 injury with their quantitative analysis (N = 5). **(F, G)** The F4/80+ kupffer cells were compared for their phagocytic activity post-BMT (N=5). **(F)** F4/80+ staining for liver kupffer cells is shown by IHC and its quantification (N = 5). **(F)** Liver kupffer cells were compared for their phagocytosis based on their phagocytic activity at every time of CCL4 injury (N = 5). **(H–J)** The flow cytometry gating of LSK (LIN-/SCA-1+/c-Kit+) and LSK sub-population based on CD34+/- and Flt3+/- cell surface markers (LT-HSC, ST-HSC, MPPs) (N = 5 to 10). **(K)** The bar graph representing negative correlation between LT-HSC and MPPs pool during disease progression. **(L)** The MSC were analyzed based on their stemness to form colonies (CFU-F) per million of BM-MNCs (N = 5). **(M)** The graph showing the positive correlation between CFU-F and LT-HSC. Arrows marking the key pathways related to innate immune responses, cell cycle and energy metabolism. Images were taken with an EVOS@FL microscope and quantified using ImageJ. Mean ± SEM; *p<0.05, **p<0.01 and ****p<0.0001. (N=40). "ns" stands for non-significant.

To assess the temporal changes in liver injury, regeneration, and the BM-SC population, C57BL/6 mice were subjected to increasing i.p. doses of CCl_4_ until week 15, at which time ascites becomes evident (a feature of decompensated cirrhosis). Samples were collected after 3, 6, 10, and 15 weeks of CCl_4_ treatment (**Figure 1A**). Biochemical analysis revealed a progressive increase in AST and ALT levels till week 15 (**Supplementary Figure 1A**), suggesting progressive liver injury. Between week 10 and 15, significant increases in total bilirubin and ammonia were observed (**Figure 1B**), and ascitic fluid exhibited a serum-ascites albumin gradient (SAAG) higher than 1.1 g/dl (**Supplementary Figure 1B**), indicating the presence of decompensated cirrhosis. There was a 34.9% increase in portal pressure (PP) by week 10 in the CCl_4_ group compared to the control group, which further increased by 26.3% at week 15 (**Supplementary Figure 1C**). These findings suggested liver failure and the development of non-acute decompensation by week 15 of chronic CCl_4_ injury.

The gross anatomy of the dissected liver showed a change from a micronodular (week 10) to a macronodular surface (week 15) (**Supplementary Figure 1D**). Histopathological analysis of liver tissue revealed inflammation, steatosis (**Supplementary Figure 1E**), mild portal fibrosis at week 3, bridging fibrosis at week 6, and cirrhosis from weeks 10 to 15 (**Figure 1C**, **Supplementary Figure 1E**). There was a progressive increase in fibrosis compared to that in the controls, as evidenced by changes in MT-, SR-, collagen-1-, and ά-SMA-positive areas until week 10. However, from weeks 10 to 15, the extent of fibrosis was comparable (**Figure 1C**, **Supplementary Figure 2A**). Interestingly, we observed a significant increase in TUNEL+ hepatocytes at week 15; however, this effect was confined to immune cells near the fibrotic septa at weeks 3, 6, and 10 (**Figure 1D**, **Supplementary Figure 2B**). Further analysis of PCNA+ hepatocytes revealed a progressive increase in hepatic regeneration until week 10. However, from weeks 10 to 15, there was a significant reduction in the number of PCNA+ hepatocytes (**Figure 1E**). Clearance of dying cells and invading gut/systemic bacteria/bacterial product by liver macrophage plays an important role in the resolution of liver injury. Both IHC and FACS analysis of liver tissue showed progressive decline in number and phagocytic function of F4/80+ liver macrophages (**Figures 1F, G**, **Supplementary Figure 2C**) after weeks of chronic liver injury. This suggests that while the progressive increase in liver fibrosis accounts for the development of cirrhosis, the increase in hepatocyte death with regeneration failure underlies the transition from compensated to decompensated cirrhosis during chronic liver injury. Loss of hepatic macrophage number and its phagocytic function begins prior to this transition.

To explore the kinetic changes in the BM reserve, we investigated the changes in BM histology and the distribution of hematopoietic stem and progenitor cells (HSPCs) in the same group of animals. In control animals, BM histology was preserved until week 15, and animals treated with CCl_4_ showed progressive increases in the loss of bone trabeculae and fat accumulation from week 6 onwards (**Supplementary Figure 3A**). FACS analysis of BM mononuclear cells (BM-MNCs) revealed a progressive increase in the number of HSPCs, which were defined as LSKs (HSPCs, LIN^-^/c-kit^+^/SCA-1^+^), with the progression of chronic liver injury compared to that in age-matched healthy controls (**Figure 1H**, **Supplementary Figure 3B**). Further examination of the LSK subpopulation, including long-term HSCs (LT-HSCs or LT-LSK; LSK/CD34^-^/FLT3^-^), short-term HSCs (ST-HSCs or ST-LSK; LSK/CD34^+^/FLT3^-^), and multipotent progenitors (MPPs; LSK/CD34^+^/FLT3^+^), revealed a progressive increase in MPPs and a decrease in LT-HSCs from week 6 onwards in animals with chronic injury compared to the controls, and no significant difference was detected in the ST-HSCs population, except for a slight increase at week 3 (**Figures 1I, J**). Interestingly, between weeks 6 and 10, LT-HSCs were reduced by more than 60%, and there was a significant decrease in the LT-HSC: MPP ratio (**Figure 1K**). Animals with CCl_4_ injury also showed progressive increases in LSKs and the loss of LT-HSCs in the blood and liver, similar to BM (**Supplementary Figures 3C, D**). Taken together, these data suggest that an increase in the LSK pool after six weeks of chronic injury was caused by the expansion of MPPs at the expense of LT-HSCs. This highlighted the disturbed balance between HSC self-renewal and differentiation, which is required to maintain demand-driven hematopoiesis in response to injury or infection (17). Furthermore, to determine whether the observed loss of LT-HSCs in these animals was due to liver injury or CCl_4_-induced injury to the BM, we compared the changes in BM LT-HSCs in thioacetamide-induced chronic liver injury (**Supplementary Figure 3E**). Similar to CCl_4_-induced chronic liver injury, in animals with thioacetamide-induced injury, the number of BM LT-HSCs was significantly lower than that in the controls (**Supplementary Figure 3F**). It was also reported that exposure to a high-fat diet decreases the percentage of LT-HSC and increases the percentage of MPP compared to a normal diet in the BM (33). Hence, our results align with that there is a loss of LT-HSC during cirrhosis.

BM mesenchymal stem cells (MSCs) play a crucial role in maintaining HSC self-renewal (34, 35). To investigate the underlying cause of LT-HSCs loss, we analyzed the changes in the numbers of Nestin+ MSCs and CFU-F colonies as a readout of the number of MSCs. Immunohistochemical analysis of Nestin+ cells in BM tissue and flow cytometric analysis of Ter119^-^CD45^-^Cd31^-^Nestin+ cells revealed a significant increase in Nestin+ MSCs at week 6 and a decrease at weeks 10 and 15 compared to those in the control group (**Supplementary Figures 4A, B**). Next, we analyzed the CFU-F colonies of BM cells as a readout of the stemness properties of MSCs. Unlike the number of Nestin+ cells, the number of CFU-F colonies in CCl_4_-treated animals significantly decreased from week 6 onwards (r=0.9573, p=0.01), which correlated with the loss of LT-HSCs (**Figure 1L, M**), suggesting the role of MSC dysfunction in the loss of LT-HSCs during chronic liver injury. Overall, our findings demonstrated the progressive loss of BM-MSCs (between weeks 3–6), followed by the loss of LT-HSCs (between weeks 6–10), regeneration failure, and the development of decompensated cirrhosis (between weeks 10–15) during the progression of chronic liver injury.

### Intra-BM infusion of syngeneic hBM cells induces the repopulation of native LT-HSCs

Exogenous infusion of hBM cells has been shown to induce functional recovery after BM dysfunction (36), and it is routinely used in clinics to manage various hematological dysfunctions (37). Thus, we next explored whether exogenous administration of BM cells could restore LT-HSCs during cirrhosis. Since immune function in cirrhotic animals is already compromised and LT-HSCs reserves are poor, we first identified the appropriate route for maximum cell delivery in cirrhotic animals: intravenous (IV) injection or direct delivery to the BM cavity by intrafemoral (IF) injection without any preparative regimen (**Supplementary Figure 5A**). Compared to the IV group, the IF group showed significantly more recruitment of injected cells to the BM and liver (**Supplementary Figure 5B**). Without a preparative regimen, IF showed more recruitment of donor BM-MNCs in the cirrhotic recipient BM; therefore, IF was selected for cell administration.

**Figure 2:**
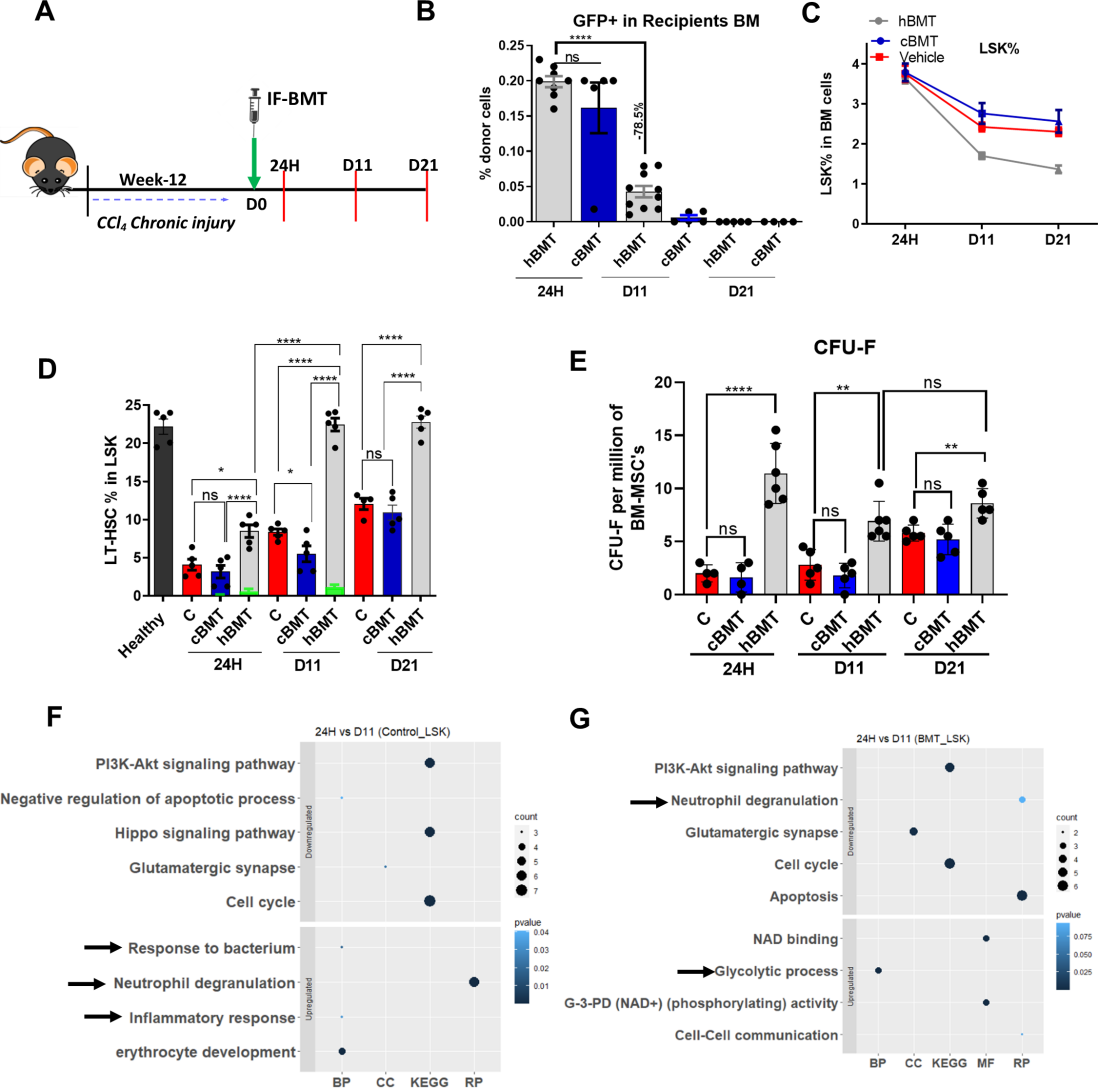
Intra-BM infusion of syngeneic healthy BM cells induces repopulation of native LT-HSC. **(A)** Schematic representation of intra-femoral (IF) infusion of healthy BM cells and points of sacrifice. **(B)** The bar graph showing the engraftment of the donor (hBMT and cBMT) GFP+ cells in recipient BM cells (N = 8 to 10). **(C)** The percentage change in LSK population was compared between hBMT, cBMT and controls (vehicle control) mice (N = 5 to 8). **(D)** The percentage change in LT-HSC at 24H, D11 and D21 post-BMT of recipients’ BM cells, while the green bar showing the donor-derived LT-HSC% in recipients’ BM LT-HSCs (N = 5). **(E)** The stemness of MSC was compared for CFU-F per million BM-MSCs at 24H, D11 and D21 post-BMT (N = 5 to 8). **(F, G)** Bubble or dot plot showing the expression of up-regulated and down-regulated pathways of BM-sorted LSK cells from BMT and a control set of mice based on biological processes (BP), cellular components (CC), Reactome pathways (RP), and KEGG pathways; and compared between 24H vs. D11 in both groups (N = 3). Size corresponds to counts, and color shows value. The data were compared based on -1<log2FC>1, P < 0.05 for their significant expression. Mean ± SEM; *p<0.05, **p<0.01 and ****p<0.0001. (N=43). "ns" stands for non-significant.

After 12-weeks of CCl_4_-injury (grade 2/3 cirrhosis), the animals were divided into three groups: Group 1 received healthy BM-cells (hBMT group), Group 2 received cirrhotic BM cells (cBMT groups), and Group 3 was the vehicle control group. hBMT and cBMT cells were administered via IF injection. CCl_4_ administration was stopped to allow natural recovery. The animals were sacrificed at 24H and on D11 and D21 (**Figure 2A**). Flow cytometry showed that the percentage of GFP+ donor cells was comparable between the cBMT and hBMT groups at 24H post-infusion; however, more than 75% of healthy and cirrhotic donor cells were cleared by D11 and were undetectable by D21 (**Figure 2B**). Further examination of BM cells showed a significantly reduction in LSK population in hBMT compared to the cBMT and control groups (**Figure 2C**). Animals in the hBMT group showed significant increases in LT-HSCs compared to those in the control or cBMT groups at 24H, which further increased and reached the levels of healthy animals by D11 and D21 (**Figure 2D**). In the hBMT group, out of the total LT-HSC population, only 0.5% at 24H and 1.2% at D11 were GFP+ LT-HSCs; however, this population was undetectable at D21 (**Figure 2D**), suggesting that the increase in BM LT-HSCs after therapy mainly involved an increase in native LT-HSCs. CFU-F analysis of total BM-MNCs revealed a significant increase at 24H, D11 and D21 in the hBMT group compared to that in the control and cBMT groups (**Figure 2E**). Unlike LT-HSCs, more than 50% of CFU-F colonies in animals treated with hBMTs were GFP+ cells at 24H, which decreased to 20% by D11 and 10% by D21 (**Supplementary Figures 5C, D**). Initial examination of the infused BM mononuclear cells revealed significantly lower numbers of LT-HSCs and monocytes, and higher numbers of MPPs and neutrophils in cirrhotic animals than in healthy controls (**Supplementary Figure 6A**). To gain insight into the mechanisms underlying the increased repopulation of native LT-HSCs following the infusion of hBM, we compared the changes in the global protein profiles of sorted native HSPCs (GFP(-)-LSK) from 24Hto D11 in control and hBMT animals (**Supplementary Figure 6B**). The differentially expressed proteins (DEP, log2FC>1, p<0.05; **Supplementary Figure 6C**) of HSPCs were examined, and there were significant increases in proteins associated with neutrophil-mediated inflammation and bacterial response in control animals (**Figure 2F**). These proteins were downregulated in animals infused with healthy BM cells from 24H to D11 (**Figure 2G**). BM cells were examined and showed significant reductions in neutrophils which differed from those in control and cBMT animals (**Supplementary Figure 6D**). These findings suggested that transient recruitment of hBM cells suppressed the inflammatory response and increased the number of MSCs in the cirrhotic BM environment. This might facilitate the self-renewal of endogenous LT-HSCs following hBMT treatment.

### Restoring the BM LT-HSC reserve accelerates the regression of fibrosis and regeneration

**Figure 3:**
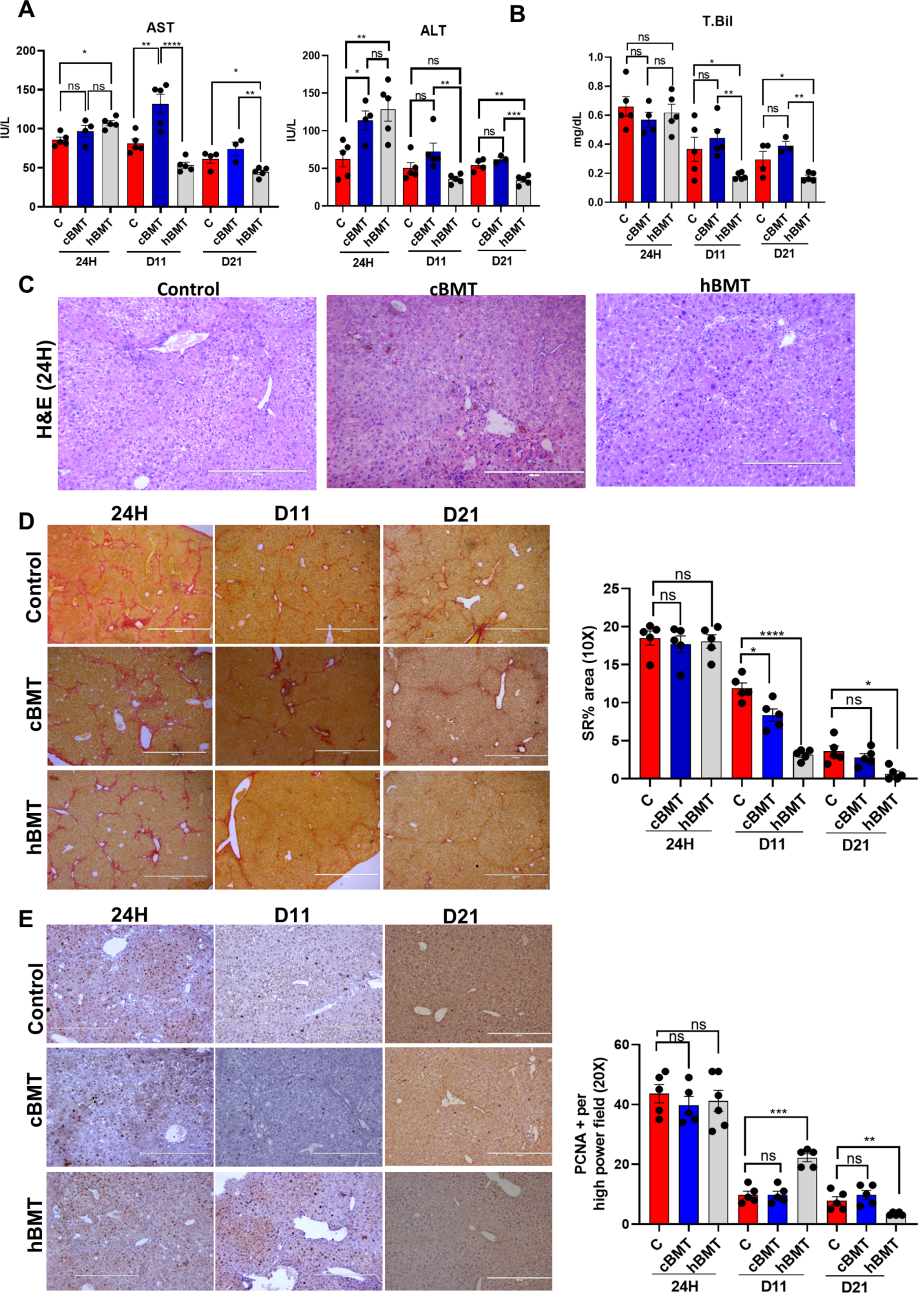
Restoring BM LT-HSC reserve accelerates regression of fibrosis and regeneration. **(A, B)** The liver injury was compared based on **(A)** AST, ALT, and **(B)** total bilirubin between the control and BMT groups (cBMT and hBMT) at 24H and D11 post-BMT. **(C)** H&E staining of liver tissue at 24H showed inflammation and necrosis between the control, cBMT and hBMT groups. **(D)** Sirius red staining showing reduced fibrosis levels from 24H, D11 and D21 between the controls and BMT groups. **(E)** Hepatocyte proliferation was checked based on PCNA+ staining on liver sections at 24H, D11 and D21 and compared at 20X blindly. Images were taken in an EVOS@FL microscope and quantified using ImageJ. Mean ± SEM; *p<0.05, **p<0.01, ***p<0.001 and ****p<0.0001. (N=20). "ns" stands for non-significant.

Next, we aimed to determine whether the restoration of BM LT-HSCs was associated with native liver repair or regeneration during cirrhosis. We compared the changes in liver injury and regeneration in the control, cBMT (with low levels of BM-LTHSCs) and hBMT (with more BM-LT-HSCs) groups during the natural recovery process at 24H, D11 and D21 after treatment (**Figure 2**). At 24H post-BMT, AST and bilirubin levels were comparable, and ALT levels in animals treated with cBMT and hBMT were significantly higher than those in control animals (**Figures 3A, B**). However, at D11 and D21 AST, ALT, and total bilirubin levels in the animals treated with hBMT were significantly lower than those in the control and cBMT groups (**Figures 3A, B**). Histological analysis of liver tissue revealed increased necro-inflammation in the cBMT and hBMT groups and cholestasis in the cBMT group compared to the control group at 24H (**Figure 3C**). Moreover, fibrosis (MT, SR, and αSMA) was comparable at 24H in all groups; however, at D11 and D21, the hBMT group showed a significant reduction in fibrosis compared to the control and cBMT groups (**Figure 3D**, **Supplementary Figures 7A, B**), suggesting increased regression of fibrosis in the presence of hBMT. After observing fibrosis regression, we examined the proliferation of hepatocytes. We found an increase in the number of PCNA+ hepatocytes on D11 following hBMT administration compared to that in the control and cBMT groups. However, this level was comparable at 24H and decreased at D21 compared to that in the control and cBMT groups (**Figure 3E**). In contrast to those subjected to hBMT, the animals subjected to cBMT not only failed to induce the repopulation of endogenous LT-HSCs but also exhibited low resolution of fibrosis and increased hepatocyte death at D11 compared to those in the control and hBMT groups (**Supplementary Figure 8A**). Interestingly, animals treated with cBMT also exhibited acute tubular necrosis (ATN) and pulmonary inflammation (**Supplementary Figure 8B**). Taken together these findings suggest that restoring the BMSC reserve accelerates the regression of fibrosis and regeneration in cirrhotic animals.

### Restoring BM LT-HSCs reserve augments innate immune function during cirrhosis

**Figure 4:**
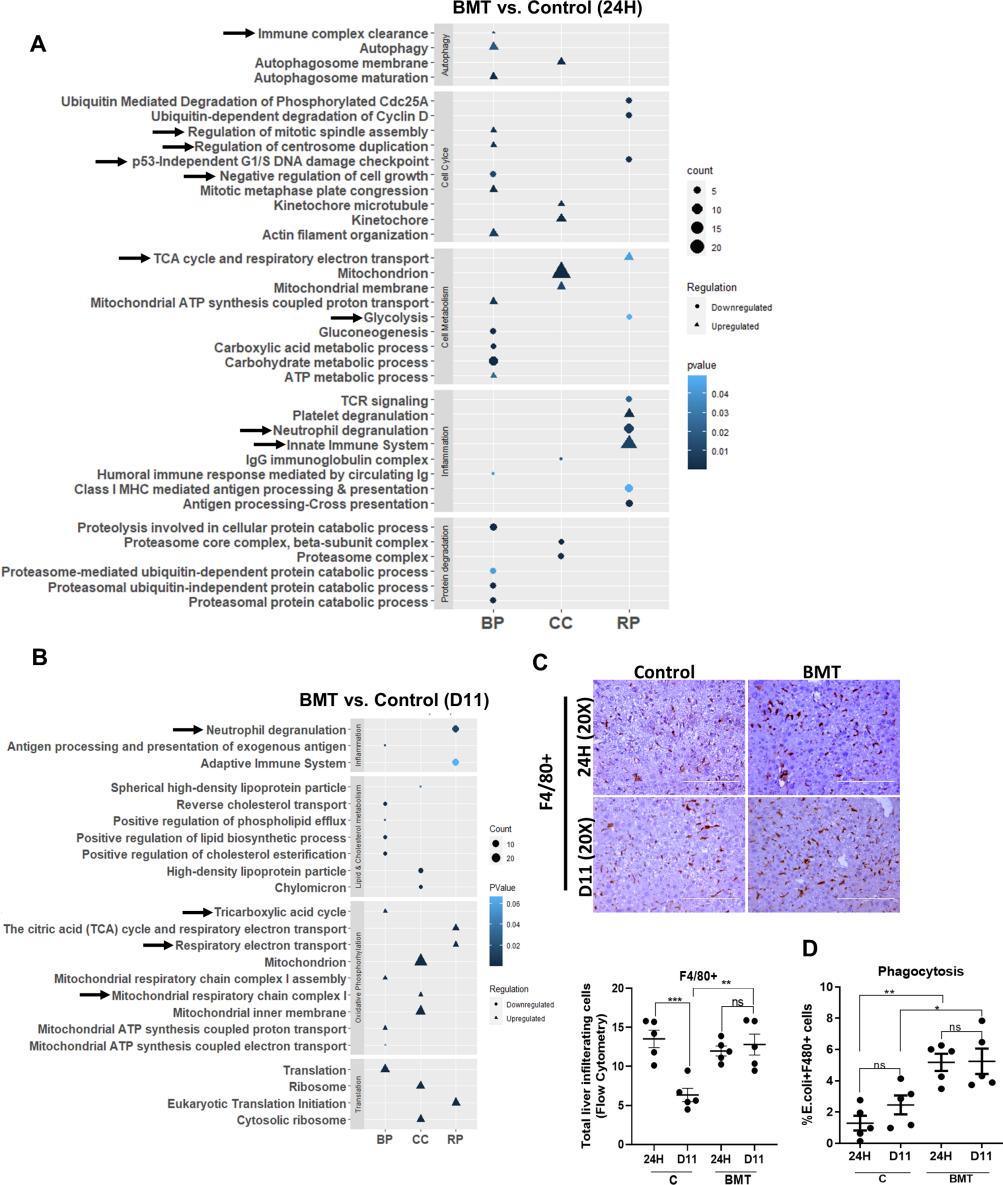
Restoring BM LT-HSC reserve augments innate immune function in cirrhosis. **(A, B)** The bubble or dot plot showing the expression of up-regulated and down-regulated pathways of BMT and control group proteins isolated from liver tissue; and compared at **(A)** 24H and **(B)** D11 post-BMT (N = 5). The data were compared based on -1<log2FC>1, p < 0.05 for their significant expression. **(C)** Representative IHC stained for F4/80+ cells post-BMT and compared between groups (control and BMT) at 24H and D11. **(D)** The graph showing the phagocytosis of F4/80+ cells at 24H and D11 and is compared between the groups. Mean ± SEM; *p<0.05, **p<0.01, ***p<0.001 and ****p<0.0001. (N=45).

To further understand the underlying cause of the increased regression of fibrosis and regeneration during BMT, we analyzed the changes in the global protein profiles of control and hBMT animal livers at 24H and D11 after BMT. Global proteomic analysis of liver tissue revealed 187 (109 upregulated and 78 downregulated) DEPs (log2FC>1.5, p< 0.05) at 24H and 186 (73 upregulated and 113 downregulated) at D11 in the BMT group compared to the control group (**Supplementary Figure 9**). Compared with those of the control group, the livers in the hBMT group exhibited significantly increased expression of proteins associated with neutrophil-mediated inflammation at 24H and D11 and increased expression of proteins associated with immune clearance and the innate immune system at 24H (**Figures 4A, B**). Flow cytometry revealed a significant reduction in the number of neutrophils in animals in the hBMT group from 24H to D11 (**Supplementary Figure 10A**). Unlike control animals where the number of F4/80+ liver macrophages significantly reduced 24H to D11, it was comparable in hBMT (**Figure 4C**). Compared to control, hBMT animals also showed significant increases in the phagocytosis and neutrophil efferocytosis function of F4/80+ liver macrophages, which are required for the effective clearance of invading pathogens and immune cells, respectively (**Figure 4D**, **Supplementary Figure 10B**). We also examined the fate of the donor-derived cells but could not find any detectable number of GFP+ cells in the liver tissue 24H after-hBMT. However, among sorted F4/80+ cells, 0.0874% (at 24H) to 0.0284% (at D11) were GFP+ (**Supplementary Figures 11A, B**). These data suggested that restoring BM-LT-HSCs during cirrhosis augments liver macrophage function and inhibits neutrophil-mediated inflammation. Our proteomic data revealed significant changes in proteins associated with glycolysis, oxidative phosphorylation, and mitochondrial energy metabolism (**Figure 4B**). Further RT-PCR analysis revealed a significant decrease in the expression of genes associated with glycolysis (*F16BP, PKM*, and *α-Enolase*) and an increase in the expression of genes associated with OXPHOS and mitochondrial energy metabolism (*PPARG, a-ketoglutarate, PDH and NRF2*) at 24Hand D11 in the liver tissue of the hBMT group compared to the control group (**Supplementary Figures 12A, B**). We observed a significant decrease in the expression of proteins associated with negative regulators of the cell cycle and an increase in the expression of proteins associated with the cell cycle at 24H (**Figure 4A**) followed by an increase in the number of PCNA-positive hepatocytes at D11 (**Figure 3E**) in the hBMT group compared to the control group. Taken together, these data showed that restoring BMLT-HSCs during cirrhosis increased liver macrophage function and inhibited neutrophil-mediated inflammation. This might contribute to the accelerated regression of fibrosis and regeneration in cirrhotic animals.

### Restoring the BM LT-HSC reserve ameliorates the progression to decompensated cirrhosis

**Figure 5:**
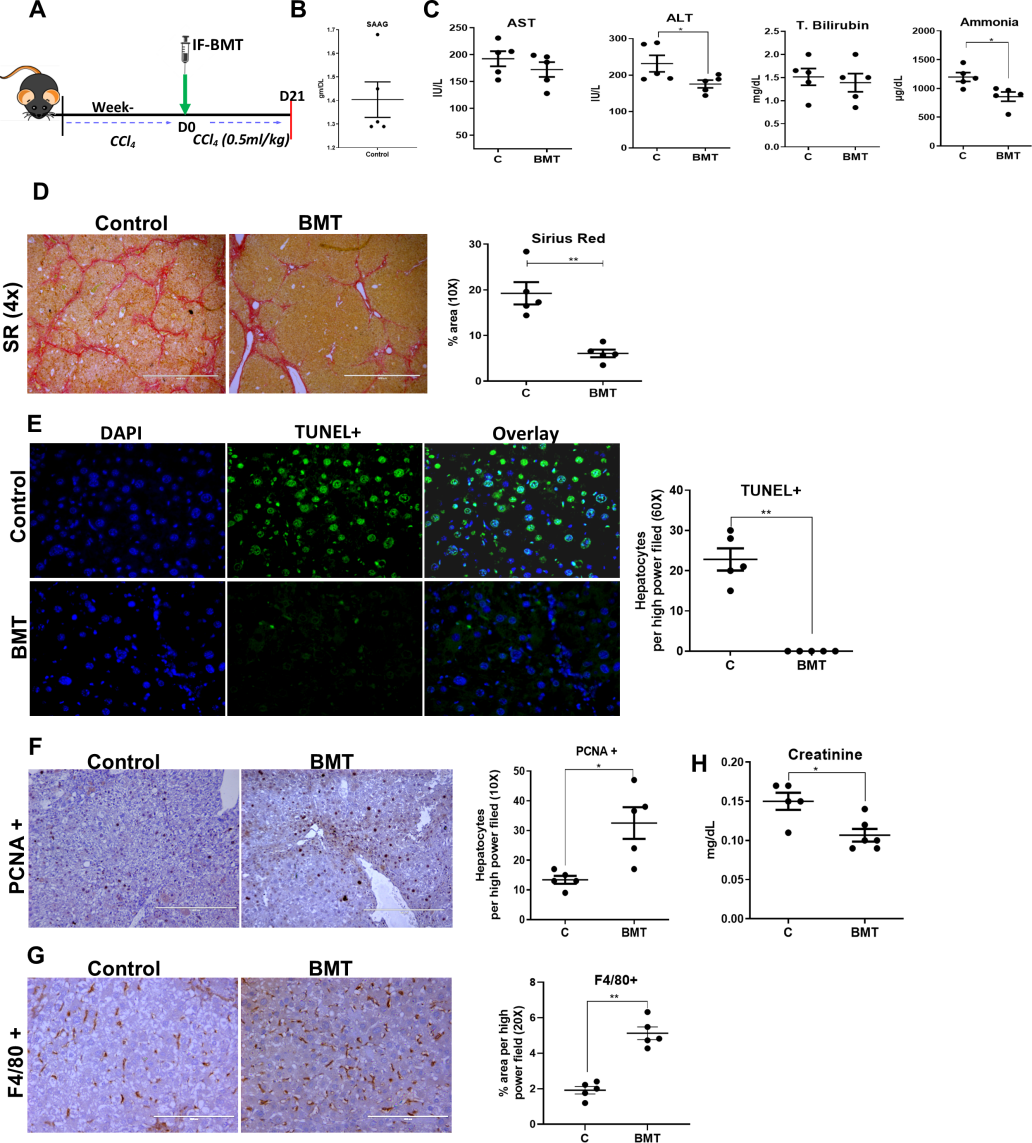
Restoring BM LT-HSC reserve ameliorates progression to decompensated cirrhosis. **(A)** The schematic representation of hBMT during the progression of hepatic decompensation. **(B)** The graphs showing the biochemical analysis of the SAAG of controls at D21 post-BMT (N = 5). **(C)** The blood biochemical test was done to compare the levels of AST, ALT, total bilirubin and ammonia post-hBMT. **(D)** The micrographs representing the fibrosis regression through Sirius red staining (4X) in BMT and control mice, and the graph showing their quantitative analysis at D21 post-BMT. **(E, F)** The micrograph (20X) showing the **(E)** TUNEL+ and **(F)** PCNA+ hepatocytes and their quantitative analysis at D21 post-BMT of treated groups in comparison to controls. **(G)** The F4/80+ staining done at D21 post-BMT (20X) between the groups. **(H)** The creatinine levels were compared to test the secondary organ damage. Images were taken in an EVOS@FL microscope and quantified using ImageJ. Mean ± SEM; *p<0.05, **p<0.01. (N=~25).

We observed a significant increase in the regression of fibrosis and regeneration with the restoration of the BMSC reserve. During chronic liver injury, we demonstrated that the loss of BM LT-HSCs occurred before regeneration failure and the development of decompensated cirrhosis. Thus, we hypothesized that restoring BM HSC reserve could prevent the progression to decompensated cirrhosis. After 12 weeks of CCl_4_-induced chronic liver injury, the animals were divided into two groups. Group 1 received an intra-BM infusion of healthy BM cells, and Group 2 received a vehicle control. CCl_4_ treatment was maintained in both groups, and the animals were allowed to develop ascites until D21 after BMT. All the animals were sacrificed on D21 after treatment (**Figure 5A**).

During this follow-up, ascites became more prominent in the control group than in the BMT group. The animals with ascites showed the presence of ascitic fluid with a SAAG >1.1 g/dl (**Figure 5B**). Compared with those in the control group, ALT and ammonia levels in the hBMT group were significantly lower at D21 after treatment (**Figure 5C**). Histopathological analysis of liver tissue revealed a significant reduction in the MT-, SR-, and a-SMA-positive areas in the hBMT group compared to the control group (**Figure 5D**, **Supplementary Figure 13**). Compared with control animals, BMT-treated animals also exhibited significant reductions in the number of TUNEL+ hepatocytes (**Figure 5E**) and increases in the numbers of PCNA+ hepatocytes (**Figure 5F**) and F4/80+ Kupffer cells (**Figure 5G**), suggesting a decrease in liver injury and increase in hepatocyte regeneration after hBMT in cirrhotic animals. We also observed a significant increase in serum creatinine levels in the controls, suggesting renal dysfunction (**Figure 5H**). Histological analysis of kidney sections revealed the presence of ATN with increased TUNEL+ renal tubular epithelial cells in the control group compared to the BMT group (**Supplementary Figure 14A**). Unlike hBMT animals, control animals also exhibited pulmonary fibrosis (**Supplementary Figure 14B**). This suggested that intra-BM infusion of healthy BMcells ameliorated the progression of liver injury, increased regeneration, and prevented the development of decompensated cirrhosis.

## Discussion

**Figure 6:**
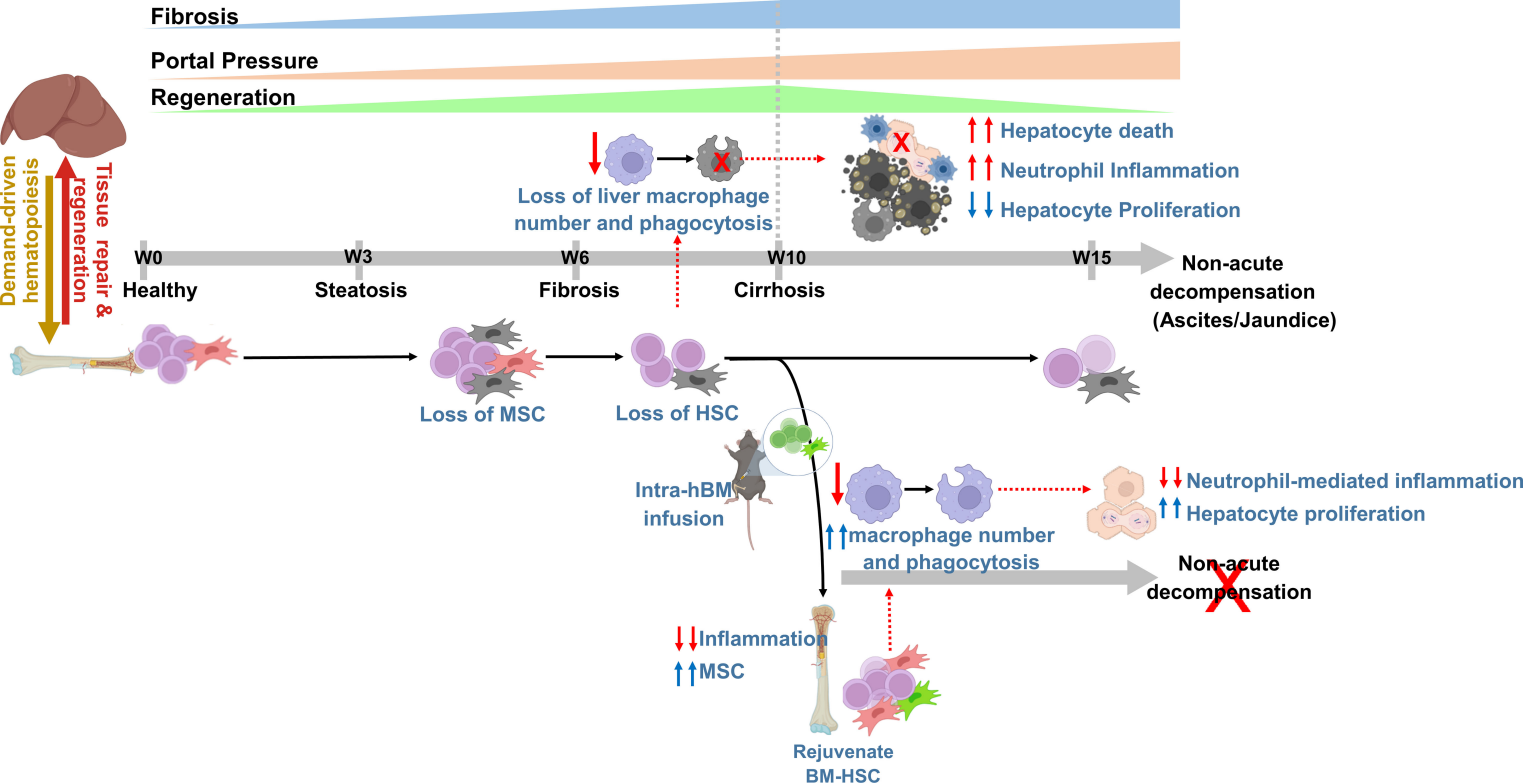
The concept diagram showing that rejuvenating bone marrow hematopoietic reserve prevents regeneration failure and hepatic decompensation in animal model of cirrhosis. The representative diagram showing progressive loss of BM hematopoietic reserve precedes the liver regeneration failure during the progression of chronic liver injury. Intra-BM infusion rejuvenates BM-stem cell reserve, augments regression of fibrosis, and regeneration, and prevents the onset of decompensated cirrhosis.

Herein we demonstrate that loss of the BM-HSC reserve preceded regeneration failure and the onset of decompensated cirrhosis. We provided the proof-of-concept that restoring the BM-HSC reserve in cirrhotic animals accelerated fibrosis regression, potentiated hepatic regeneration, and prevented the progression of non-acute decompensation (**Figure 6**).

Preventing the transition from compensated to decompensated cirrhosis is the major challenge in the management of chronic liver disease. Progressive increases in liver fibrosis and portal hypertension with aberrant hepatocyte regeneration are shown to be associated with architectural disruption and the development of cirrhosis (38, 39). An increase in portal hypertension and systemic inflammation due to liver injury and gut dysbiosis is thought to be associated with the development of decompensated cirrhosis (6, 40). Despite the reports of regeneration failure as a cause of liver failure, the kinetic change in liver regeneration in the transition from compensated to non-acute decompensated cirrhosis is not clearly defined. We observed a progressive increase in fibrosis and hepatocyte regeneration until the compensatory stage (week 10). During the transition from compensated to decompensated cirrhosis, fibrosis was comparable, but there was a further increase in PP, and a marked increase in hepatocyte death without compensatory hepatocyte proliferation (from weeks 10–15). This highlights the contribution of increased liver injury and regeneration failure in the transition from compensated to decompensated cirrhosis in this model, similar to human cirrhosis (6, 40).

Demand-adaptive hematopoiesis plays a central role in the resolution of damage and repair (17, 18). In comparison to age-matched healthy animals, animals with chronic liver injury showed gradual increase in the number of HSPCs (BM-LSK) with disease progression, indicating demand-adapted hematopoiesis. The balance between HSC self-renewal and HSC differentiation is required to maintain demand-driven hematopoiesis in response to injury or infection (17). In the initial stage of liver injury, this balance is maintained; however, during the transition from fibrosis (week 6) to cirrhosis (week 10), this balance is disrupted, and a further increase in the LSK pool is mainly attributed to the expansion of MPPs at the expense of LT-HSCs. Loss of BM-HSCs has also been reported in an animal model of high-fat diet-induced liver injury (28) and patients with advanced liver cirrhosis (22).

BM-MSCs play an essential role in the maintenance of HSC self-renewal (34, 35), and we observed a significant loss of MSC colonies from week three onwards before the loss of LT-HSCs. The CFU-F ability of BM-MSCs but not the number of Nestin+ MSCs was proportional to the number of BM-LT-HSCs. Cellular and functional exhaustion of BM-MSCs had been shown to be associated with the loss of HSCs in chronic liver injury[22-24,28]. Thus, it is rational to conclude that the prior loss of BM-MSCs could contribute to the loss of LT-HSCs during injury.

Interestingly, we observed that the loss of BM LT-HSCs preceded regeneration failure and the onset of decompensation in the progression of chronic liver injury. Further restoring native BM-LT-HSC reserve accelerated fibrosis regression, decreased hepatocyte death, potentiated regeneration, and prevented the transition to decompensated cirrhosis. This highlights the potential contribution of decreased BM-LT-HSC reserve in increased hepatocyte injury and regeneration failure in the transition to decompensated cirrhosis. Restoring BM LT-HSCs reserve significantly increased the proteins associated with immune clearance and decreased neutrophil-mediated inflammation in the liver. Further analysis of liver tissue showed a significant reduction in the number of neutrophils, loss of liver macrophage pool, and increased bacterial phagocytosis and neutrophil efferocytosis. In response to liver injury, BM-derived macrophages play a central role in the clearance of cellular debris, inflammation, and bacteria/bacterial products derived from gut or systemic infection (9, 16, 41). Endotoxemia and infection have been shown to inhibit liver regeneration and promote hepatocyte injury in cirrhotic animals with acute decompensation (12, 13). Increased intestinal bacterial translocation due to gut dysfunction and poor liver clearance is the hallmark of cirrhosis, particularly in the transition to decompensated cirrhosis (7, 11). BM-derived monocyte/macrophage depend entirely on BM-HSCs to meet their increased demand in response to injury and infection (17, 34). Recently *in vivo* fate-mapping in mice showed the direct contribution of BM-HSC in replenishment of liver kupffer cells (42). Our data showed that during chronic liver injury, the loss of both the liver macrophage pool and BM LT-HSCs starts before the increase in liver injury and regeneration failure. During cirrhosis, mitochondrial dysfunction leads to a shift in hepatocyte energy metabolism from oxidative phosphorylation (OXPHOS) to glycolysis (43). Our data showed that with the restoration of innate immune function, there is a shift from glycolysis to OXPHOS, the restoration of metabolic function in the liver along with the restoration of the BM reserve. Based on these observations, we propose that the loss of BM-HSCs contributes to defects in macrophage-mediated liver clearance during injury. This may result in increased hepatocyte death and regeneration failure due to compromised liver clearance of invading gut-derived infections and neutrophil-mediated inflammation during the transition from compensated to decompensated cirrhosis. In course of chronic liver injury due to repeated insults, monocyte/macrophage may develop innate immune memory, leading to functional impairment of these cells against infection (24). Our data showed significant improvement in bacterial clearance function of liver macrophage with restoration of BM-LT-HSC in cirrhotic. Whether observed improvement in bacterial clearance function of liver macrophages is due to loss of innate immune memory or not need further in-depth investigation.

Rejuvenating BM reserve has recently been shown to improve tissue repair responses in aging (44, 45). Our data showed that BM directed infusion of hBM cells induced the repopulation of native LT-HSCs in cirrhotic BM without engraftment of donor hematopoietic stem cells. It dampens the neutrophil-mediated inflammation and increased the number of functional MSCs in cirrhotic BM environment. Unlike donor LT-HSCs which negligibly contributed to the increase in BM-LT-HSC and cleared by D21, we observed significant engraftment of donor MSC in cirrhotic BM which were detectable even at D21. Unlike hBM cells, infusion of cirrhotic BM cells not only fails to induce the repopulation of native LT-HSCs, it even worsens fibrosis, which was consistent with a previous report (41). In comparison to hBM, cirrhotic BM cells showed a significantly lower number of LT-HSC, MSC, monocytes and a higher number of MPP and neutrophils. Broad defect in energy metabolism has recently been shown to be associated with functional exhaustion of bone marrow mesenchymal stem cells in cirrhotic patients (30). Together these observations suggest that, intra BM infusion of healthy BM cells suppressed the inflammatory response and increased the number of MSCs in the cirrhotic BM environment. This might facilitate the rejuvenation of endogenous LT-HSCs following hBMT. Indeed restoring BM-MSC function (34, 46) and dampening BM inflammation (47) has recently shown to rejuvenate BM hematopoietic stem cell reserve.

In the present study, the donor BM cells did not engraft and the increase in LT-HSCs was mainly attributed to the repopulation of native LT-HSCs, which reduced the risk of graft versus host response, and further study of MHC-mismatched donors is required to establish the safety of allogenic BM cell infusion for future clinical translation. Additionally, in this study, we used a heterogeneous population of BM-mononuclear cells for infusion and did not examine the cell type responsible for the induction of native LT-HSCs repopulation in the recipient. Further investigation is required to determine the underlying mechanism and establish future clinical protocols.

In summary, the current study revealed that the loss of the BM-HSC reserve accounts for the poor resolution of liver injury and regeneration failure during chronic liver injury. It also provides the proof-of-concept that rejuvenating BM-HSC reserve will accelerate the resolution of injury, prevent regeneration failure and the onset of decompensation in cirrhotic. Future work in this direction can fine-tune and develop interventional strategies to rejuvenate BM as a novel therapeutic approach to mediate the regression of liver injury and regeneration during cirrhosis.

## Data availability statement

The datasets presented in this study can be found in online repositories. The names of the repository/repositories and accession number(s) can be found below: JPST003156 and JPST003146 (JPOST).

## Ethics statement

The animal study was approved by Institutional Animal Ethics Committee Board of Institute of Liver and Biliary Sciences. The study was conducted in accordance with the local legislation and institutional requirements.

## Author contributions

NN: Writing – review & editing, Writing – original draft, Visualization, Validation, Software, Resources, Methodology, Investigation, Formal analysis, Data curation, Conceptualization. DM: Writing – review & editing, Methodology, Investigation. DK: Writing – review & editing, Methodology. ER: Writing – review & editing, Methodology. DT: Writing – review & editing, Methodology. SK: Writing – review & editing, Methodology. SD: Writing – review & editing, Methodology. MB: Writing – review & editing, Methodology. SM: Writing – review & editing, Methodology. PB: Writing – review & editing, Methodology. AK(11^th^ author): Writing – review & editing, Methodology. CB: Writing – review & editing, Methodology. SB: Writing – review & editing, Methodology. SS: Writing – review & editing, Supervision, Project administration, Funding acquisition, Conceptualization. AK(15^th^ author): Writing – review & editing, Writing – original draft, Visualization, Supervision, Project administration, Funding acquisition, Conceptualization.
