# Supplementary material for: Rejuvenating bone marrow hematopoietic reserve prevents regeneration failure and hepatic decompensation in animal model of cirrhosis
Source: Front Immunol. 2024 Aug 12;15:1439510. doi: 10.3389/fimmu.2024.1439510 (PMC11345600; doi:10.3389/fimmu.2024.1439510)
Supplement: Supplementary file 1 [file DataSheet_1.docx]

**Supplementary Data**

**Supplementary Figures:**


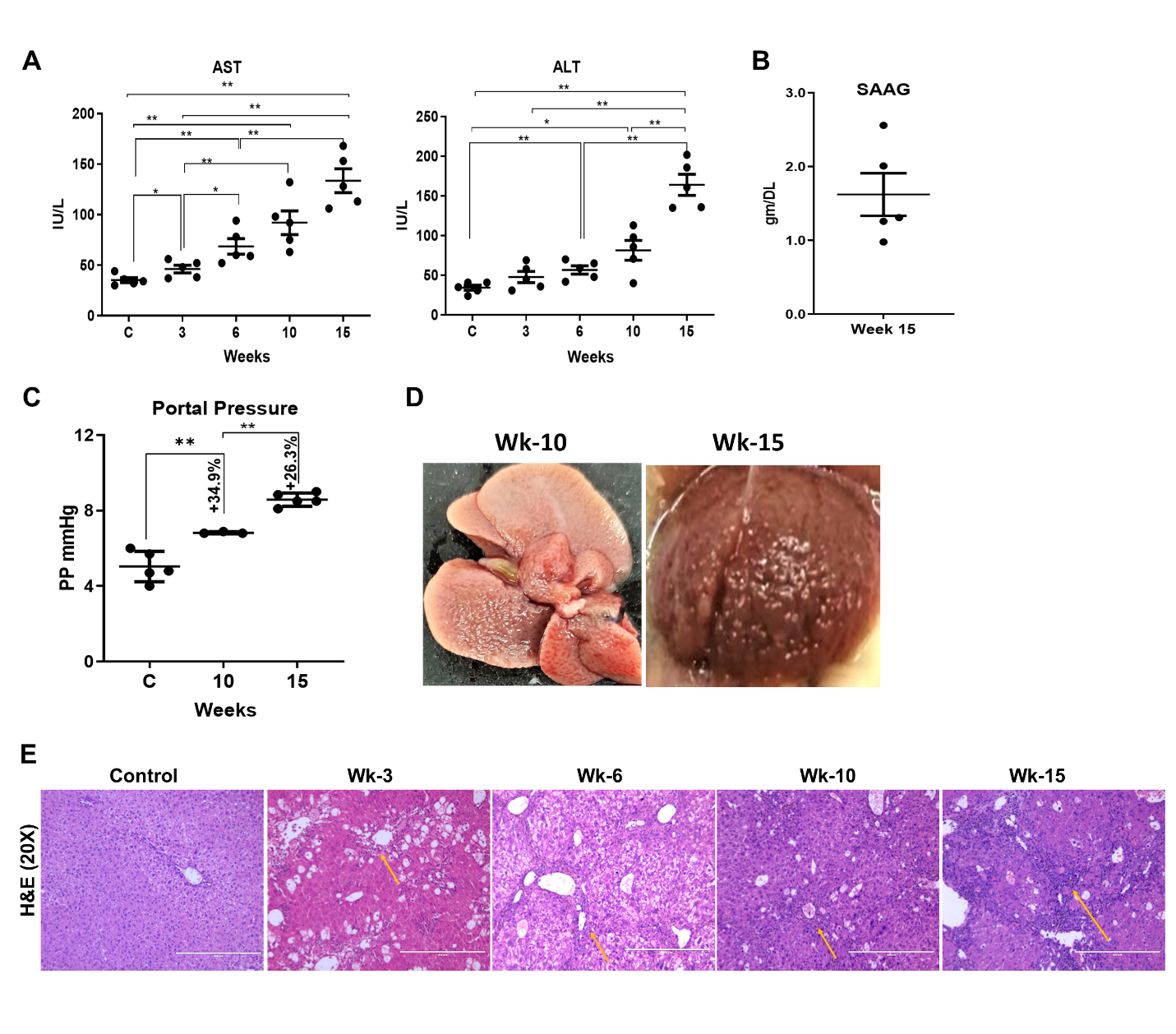
**Figure S1.** **Pathophysiological changes during progression of chronic liver disease**. (A) The graph showing the biochemical parameters of liver injury measured as rise in AST and ALT with the progression of chronic liver injury (n=5). (B) Serum ascites level measured as SAAG level post week-15 of CCl4 injury (N = 5). (C) The graph showing the hemodynamic assessment between the group of mice at week-10 and week-15, and compared with the control set of mice mice (N = 5). (D) The gross anatomy of the dissected liver showing the nodularity change from a micronodular (week-10) to a macronodular surface (week-15). (E) The micrographs showing the progressive increase in inflammation from week 6 to 15 by hematoxylin and eosin staining (H&E; 10X). The images were taken in EVOS@FL2 for different areas and quantified using ImageJ. Mean ± SEM; *p<0.05, **p<0.01, ***p<0.001 and ****p<0.0001.


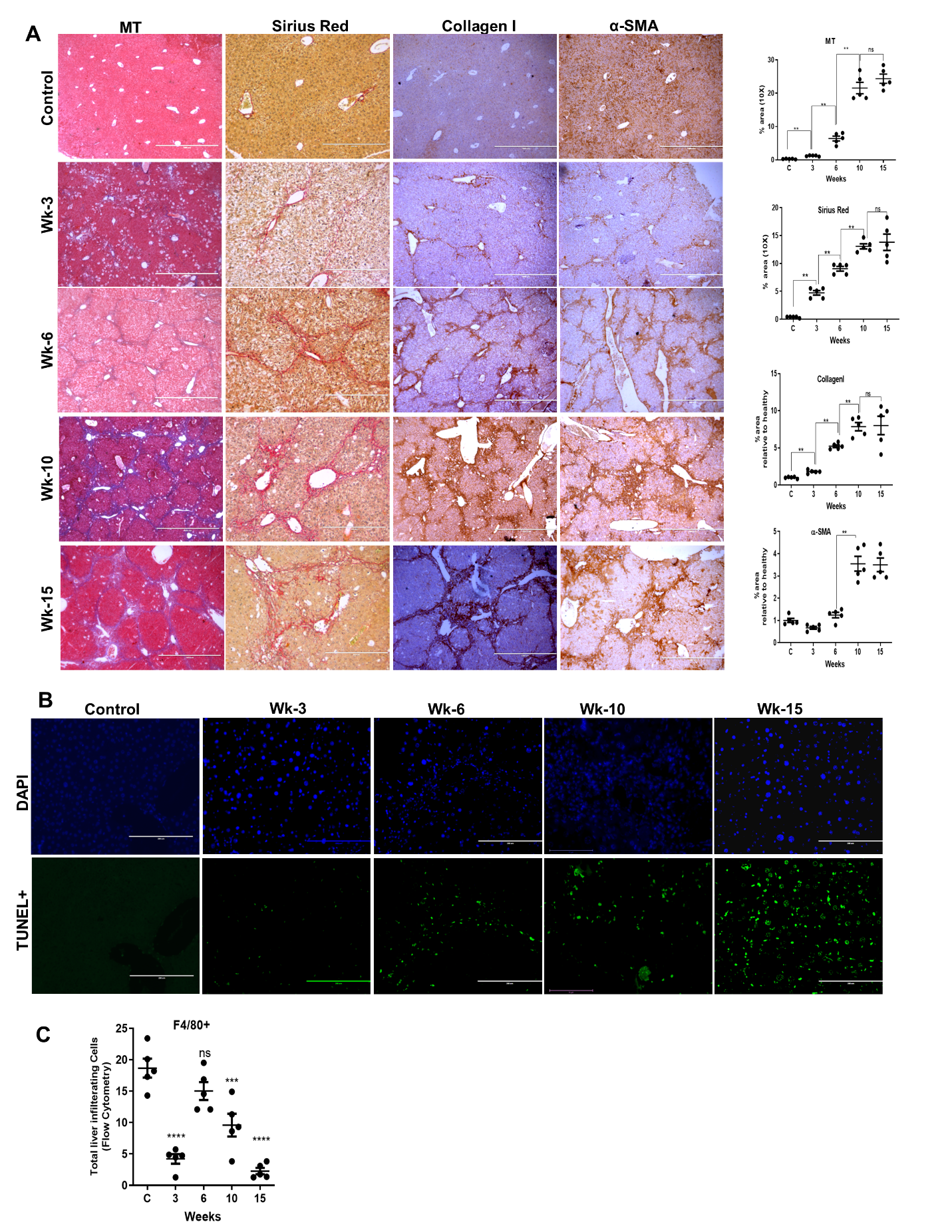
**Figure S2.** **Histopathological changes in fibrosis and cell death.** (A) The micrographs (4X) showing the levels of fibrosis which is compared based on stainings: Massons’ Trichrome (MT), Sirius red, Collagen-1 and α–SMA and compared based on their quantitative analysis (N=5). (B) The cell death analysis was performed by in-situ labeling of apoptosis-induced DNA strand breaks (TUNEL assay) for dead hepatocytes at every week. (C) The graph showing the flow cytometer analysis of F4/80+ cells from liver infiltrating cells (N=5). The images were taken in EVOS@FL2 for different areas and quantified using ImageJ. Mean ± SEM; *p<0.05, **p<0.01, ***p<0.001 and ****p<0.0001.


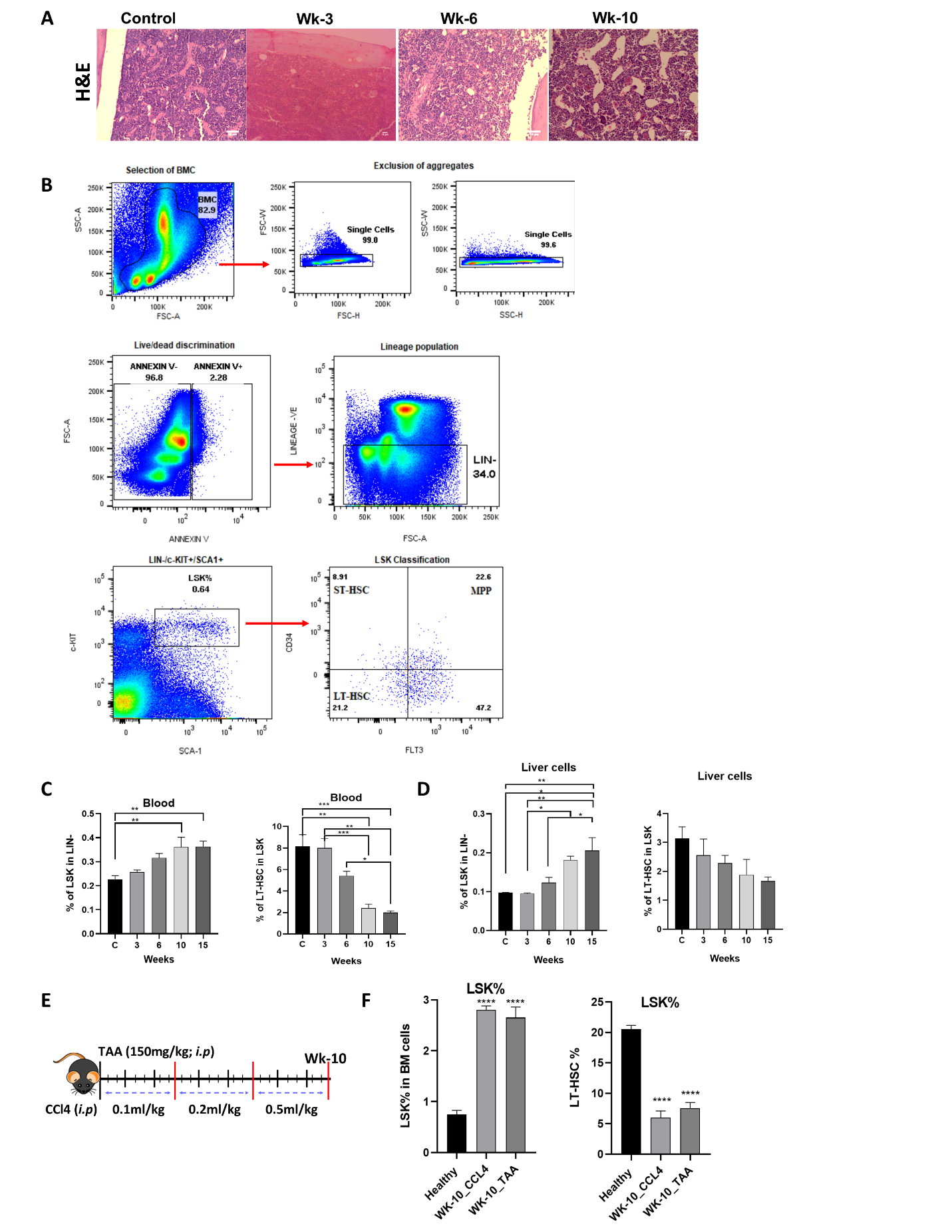


**Figure S3.** **Bone marrow changes.** (A) The micrographs showing the H&E staining of mice femur, showing the change in cellularity of BM from week 10 with loss of trabeculae (magnification 20X) with disease progression. (B) The Figure illustrates the gating strategy used for BM hematopoietic stem cells enumeration. The live dead discrimination was done using annexin V staining. (C-D) The bar graphs showing the change in LSK and LT-HSC percentage, with the progression of injury, in (C) blood and (D) liver cells (N=5). (E) Schematic representation of chronic liver injury induced by thioacetamide (150mg/kg) and CCl4 for 10 weeks. (F) The graphs showing the change in BM LSK% and LT-HSC% between thioacetamide and CCl4 induced chronic liver injury (N=5-7). The images were taken in EVOS@FL2 for different areas. Mean ± SEM; *p<0.05, **p<0.01, ***p<0.001 and ****p<0.0001.


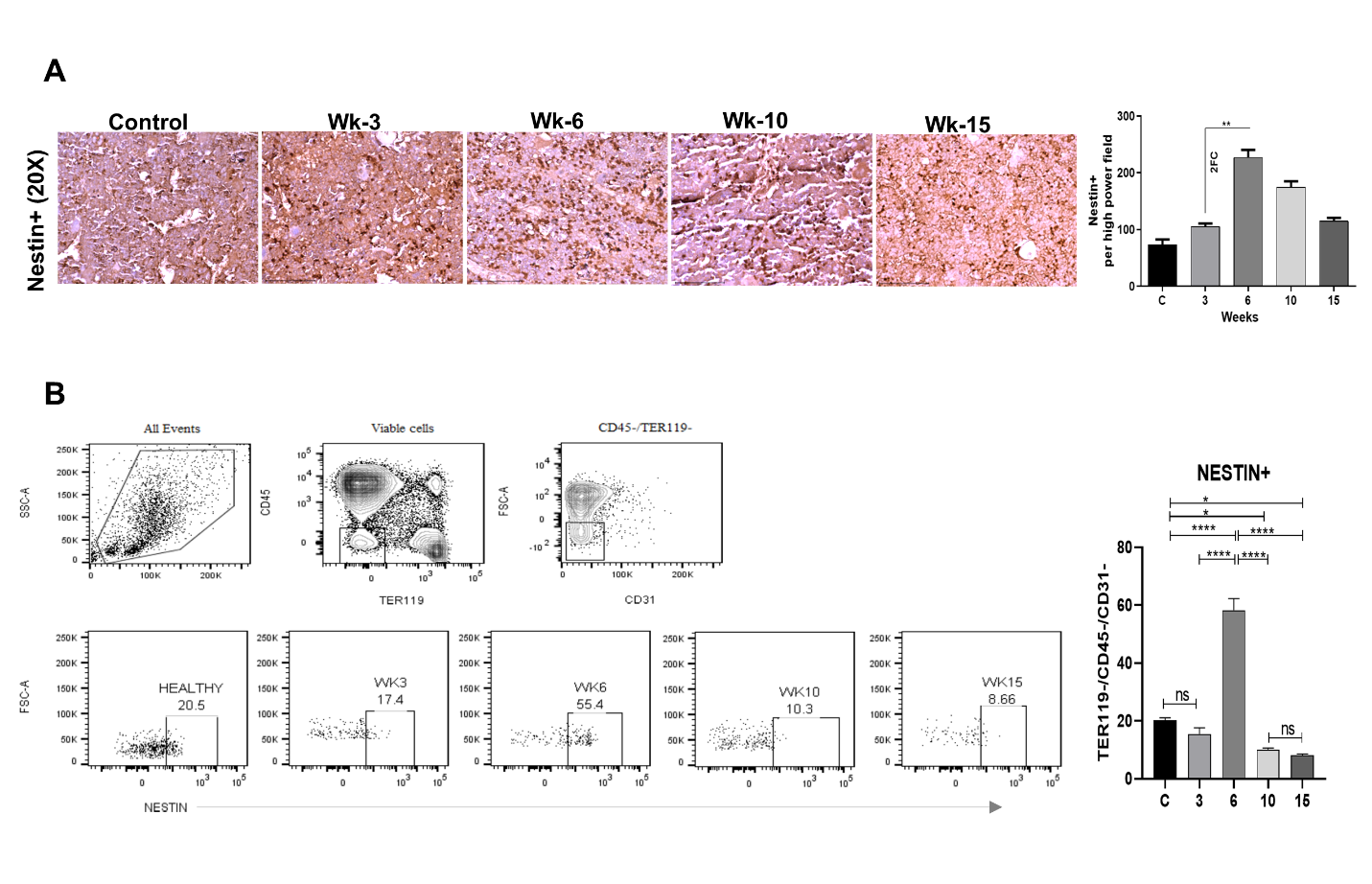
 **Figure S4: Change in BM-MSCs.** (A) The micrographs showing the immunohistochemistry for Nestin+ MSC on the BM sections (>2.5µm) and quantified for Nestin+ area using imageJ (20X). (B) Further, Nestin+ MSC was quantified based on cell surface markers (TNC;Nestin+) during chronic liver injury. The images were taken in EVOS@FL2 for different areas. Mean ± SEM; *p<0.05, **p<0.01, ***p<0.001 and ****p<0.0001


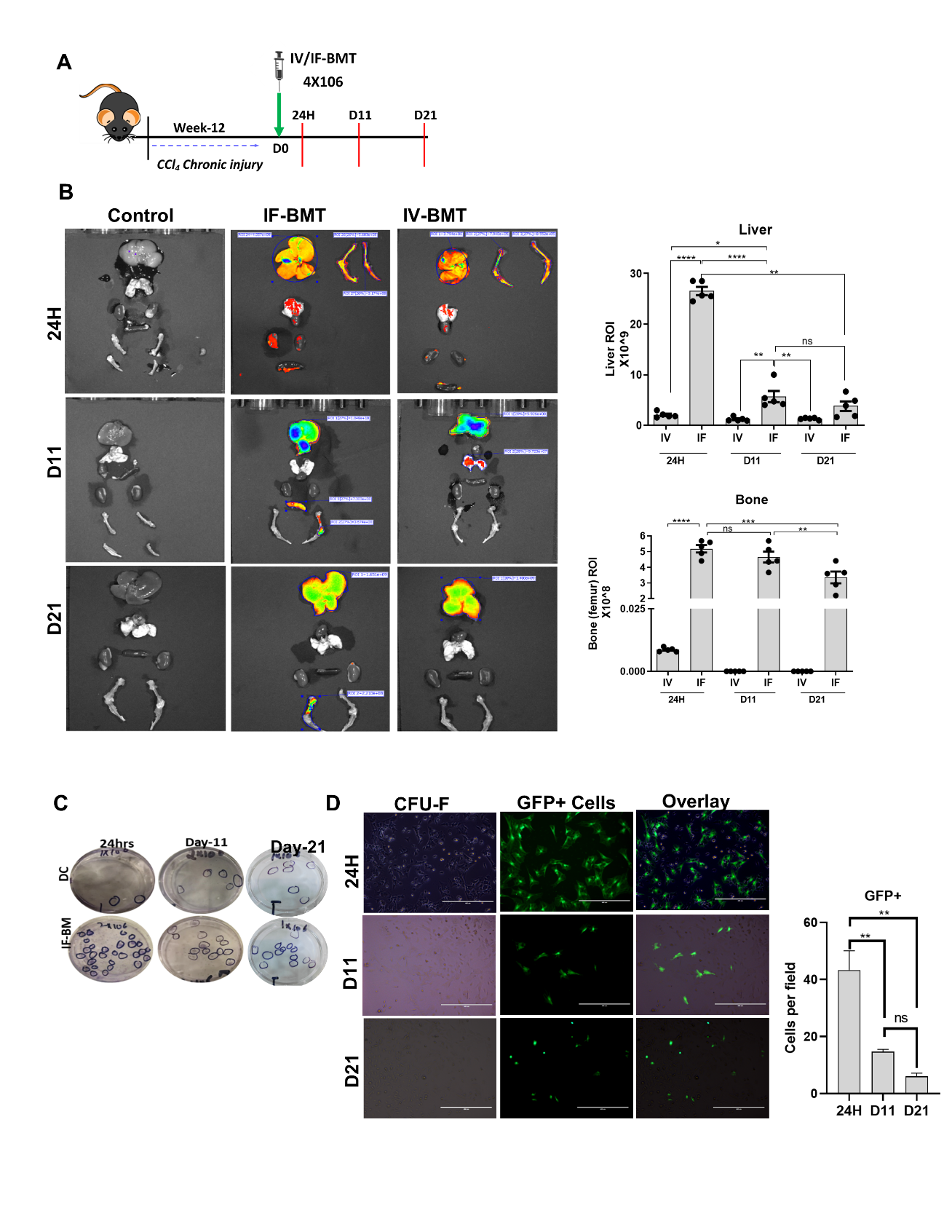


**Figure S5. Cells localization post cell therapy.** (A) The schematic representation of mode of cell therapy: IF and IV, where the two modes of BMT compared for D21 in both the groups and compared with the control. (B) The cells localization was compared based on ex-vivo imaging through IVIS data: control, IF and IV for 24H, D11 and D21. The images quantified based on ROI for liver and bones (femur and tibia) for every time interval (N=5). (C) The images showing the number of colonies formed at 24H, D11 and D21 post BMT. (D) The images showing the colonies formed at these time points with the reduction in donor derived fibroblast cells (GFP+) from 24H to D21. Images were taken in EVOS@FL2 (20X). Mean ± SEM; *p<0.05, **p<0.01, ***p<0.001 and ****p<0.0001.


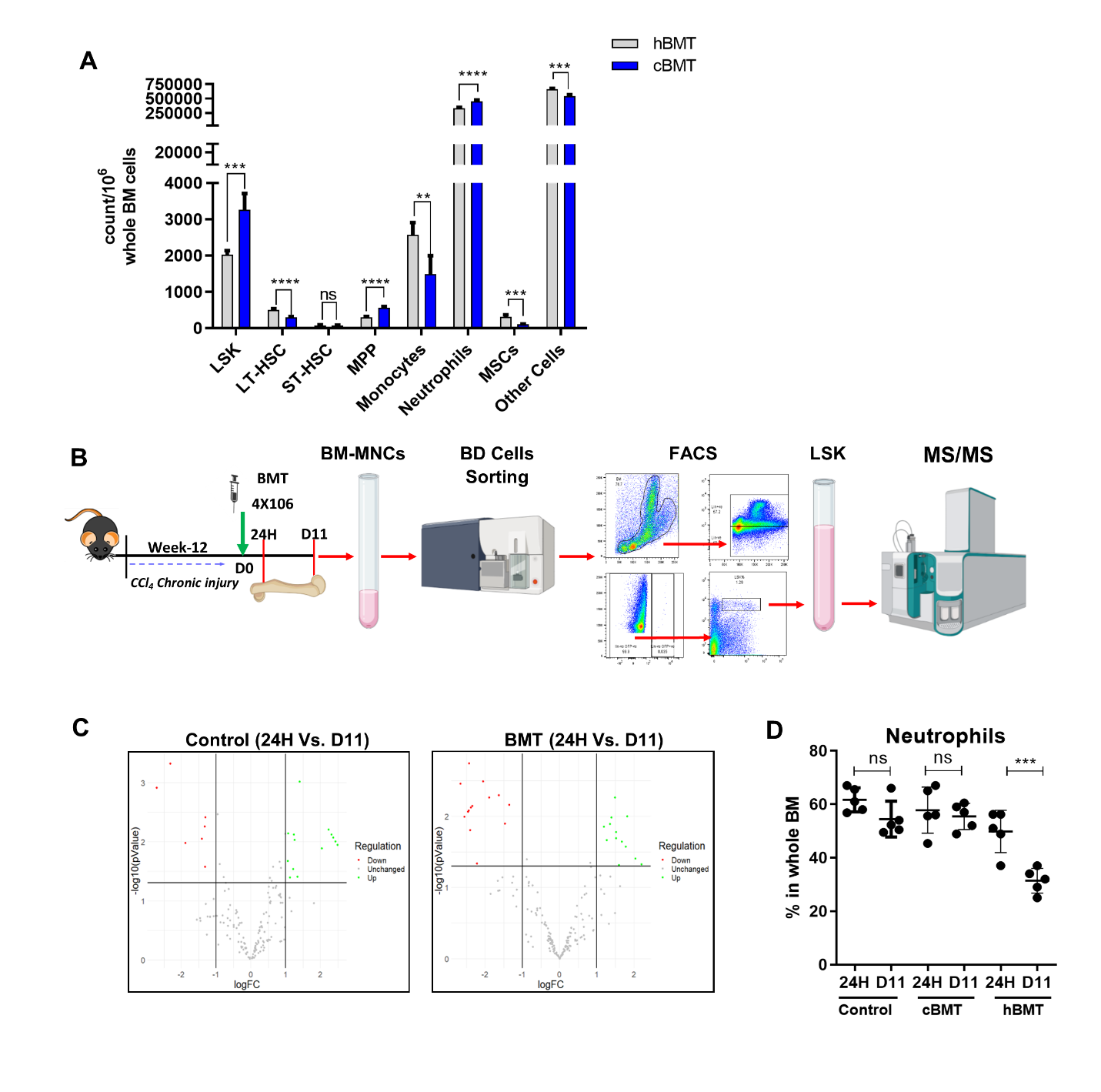


**Figure S6: Cells distribution.** (A) The bar graph showing the change in counts for types of cells between hBMT and cBMT post-therapy. (B) The schematic representation of steps followed for mass spectrophotometry. (C) Volcano plot showing the upregulated and downregulated significant proteins. The data was compared based on -1<log_2_FC>1, p< 0.05 for their significant expression. (D) The graph showing the change in neutrophils and monocytes percentage at 24H and D11 between control, cBMT and hBMT groups. Mean ± SEM; *p<0.05, **p<0.01, ***p<0.001 and ****p<0.0001.


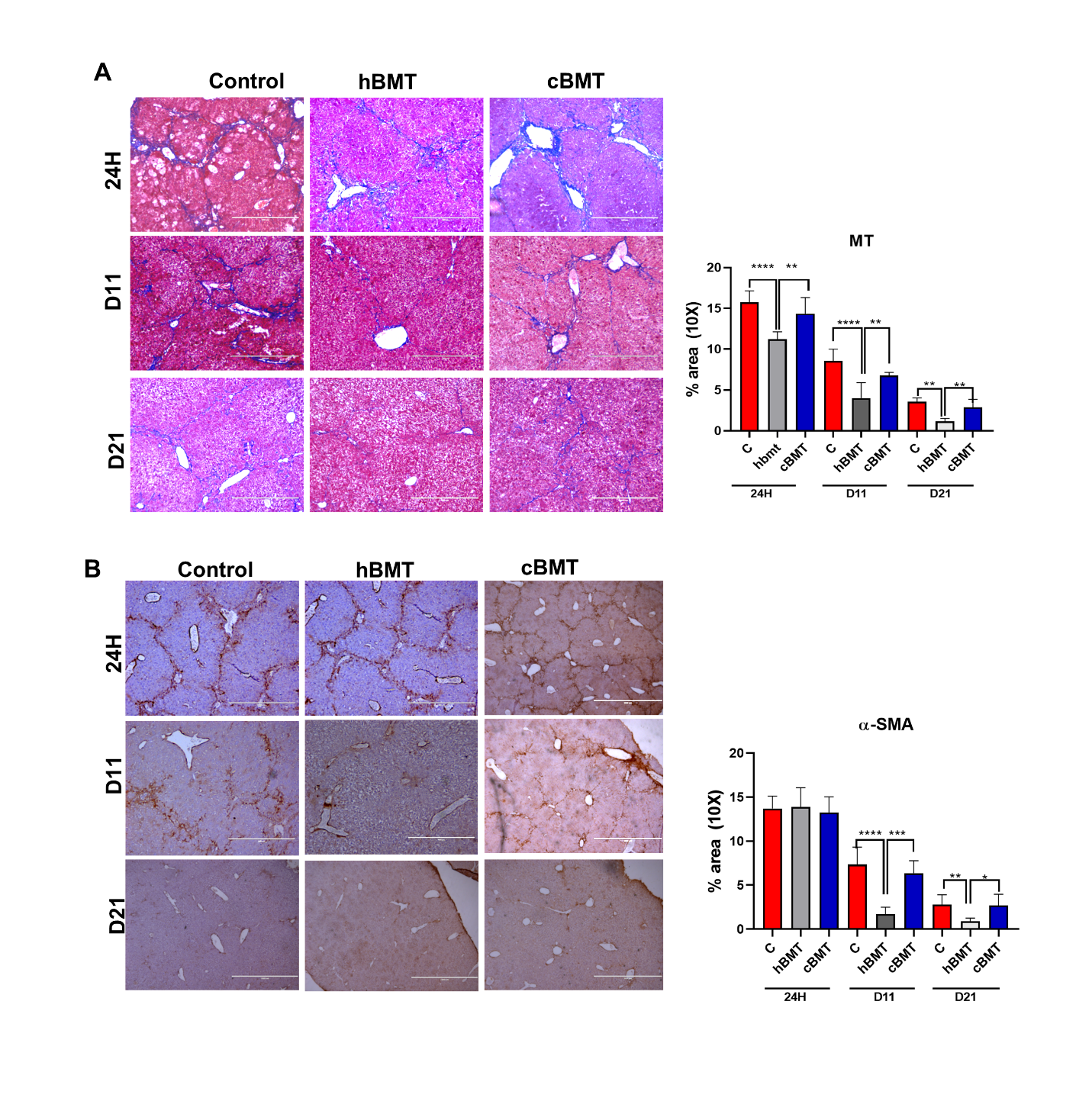


**Figure S7: Post BMT changes in liver.** (A&B) The micrographs showing the histopathology of fibrosis based on (A) MT and (B) α–SMA positive stained area post-BMT for the time points: 24H and D11. The images were taken in EVOS@FL2 at 10X maginification for different areas. Mean ± SEM; *p<0.05, **p<0.01, ***p<0.001 and ****p<0.0001.


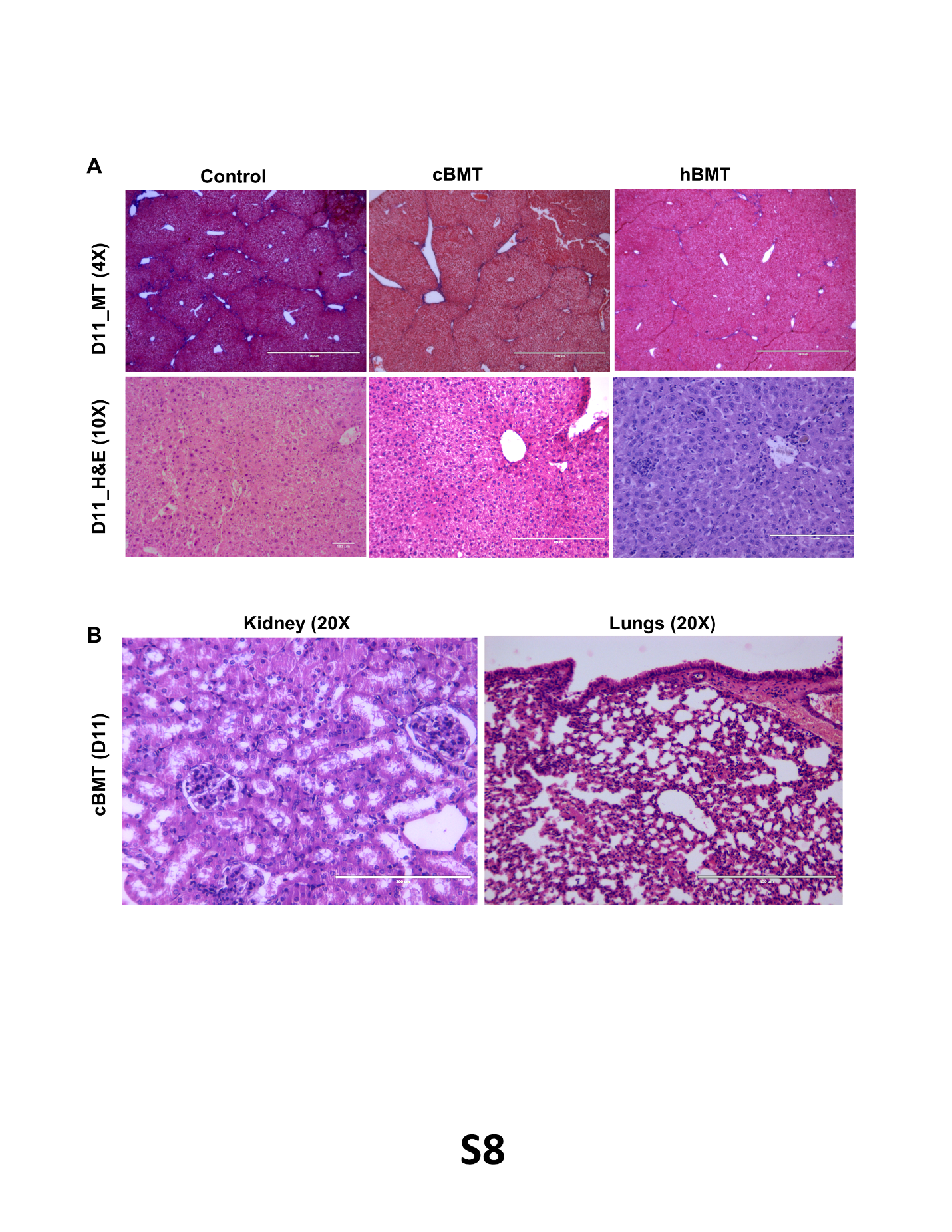


**Figure S8. Type of cells for BMT.** (A) The representative micrographs showing the fibrosis regression through MT staining in hBMT group as compared to controls and cBMT, while the cBMT showed most of the hepatocyte’s death (H&E, bottom figure) as compared to controls and hBMT (10X). (B) The micrographs showing the secondary organ damage in cBMT groups with acute tubuar necrosis and pulmonary fibrosis (20X).


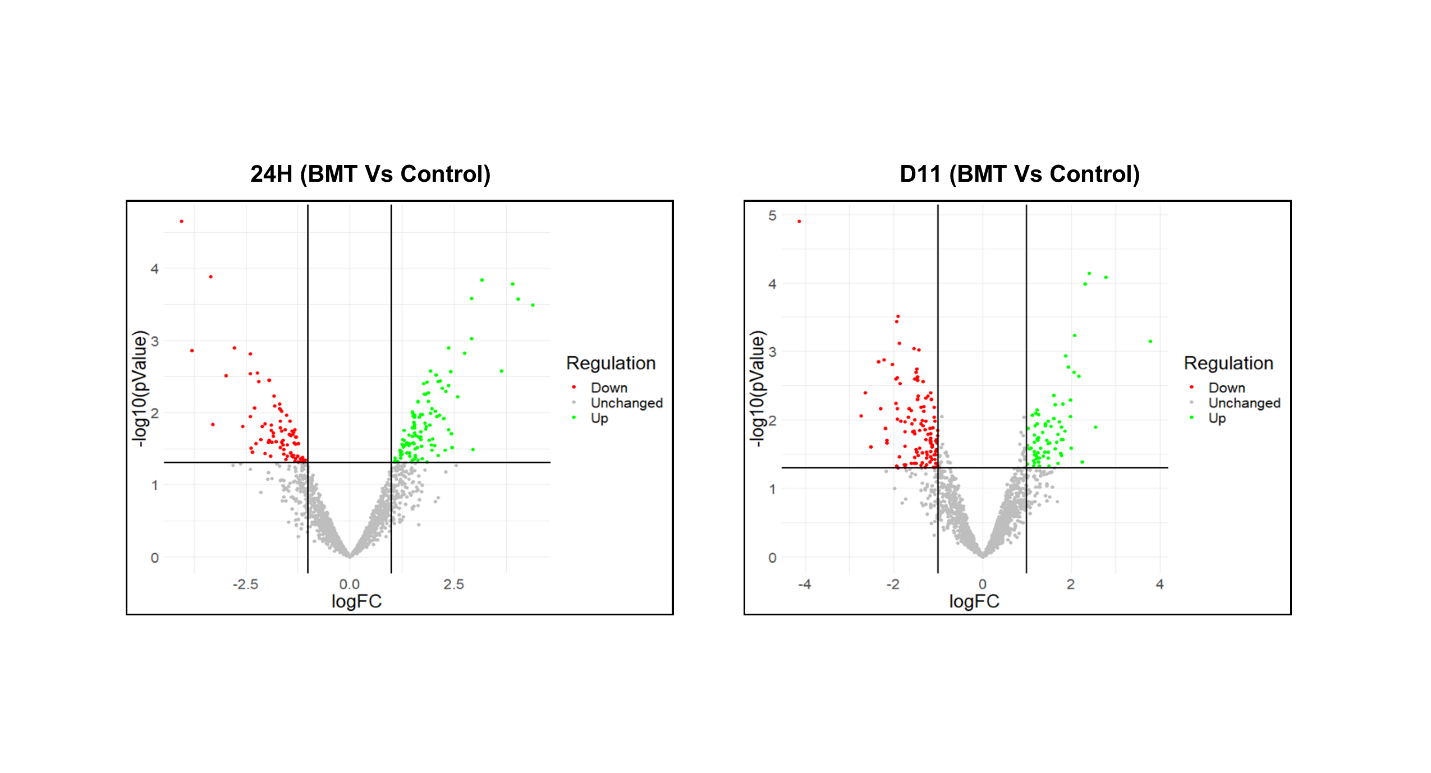


**Figure S9. Volcano plot.** Volcano plot showing the upregulated and downregulated expression of significant proteins for 24H and D11 between BMT groups and compared with control set of mice. The data was compared based on -1<log_2_FC>1, p< 0.05 for their significant expression.


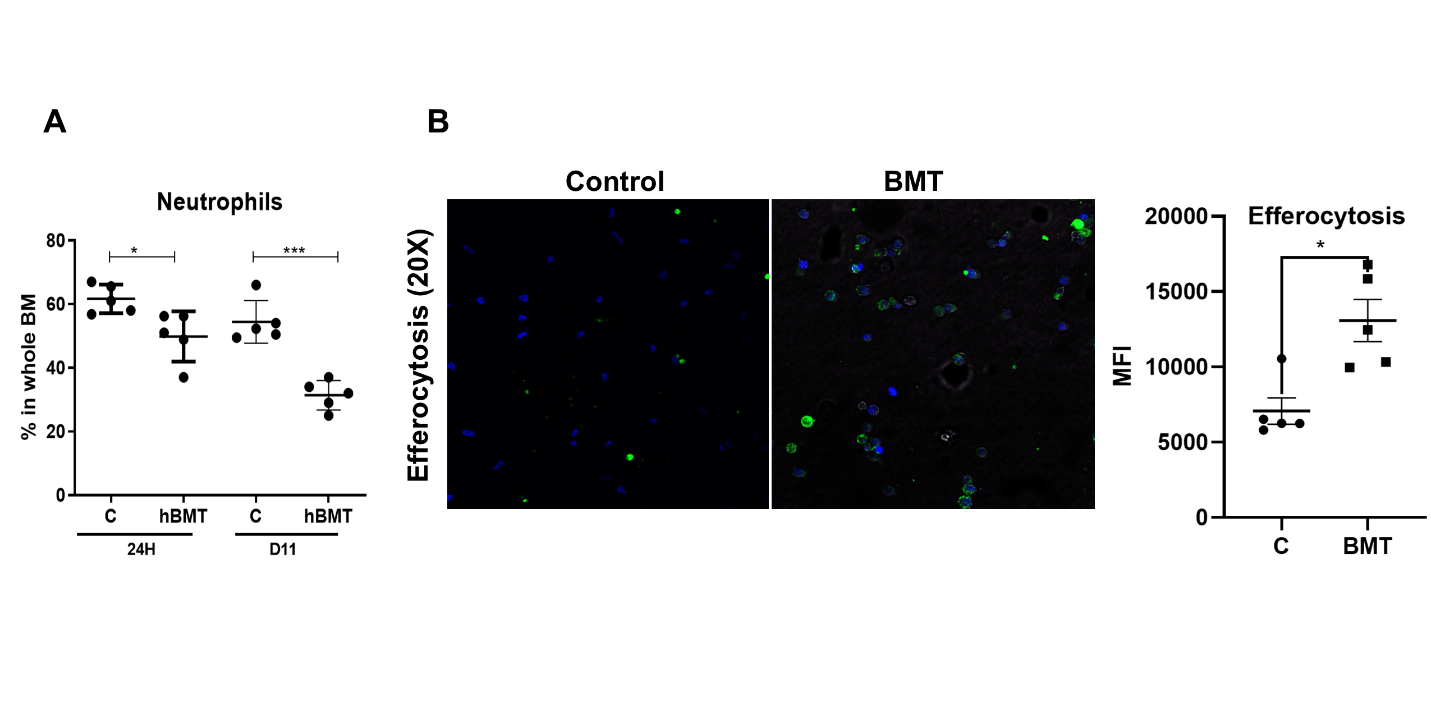


**Figure S10: Post BMT change.** (A) The graphs showing the percentage change in neutrophils between controls and hBMT at 24H and D11 post-therapy. (B) Efferocytosis was compared between control and hBMT at D11 post-therapy. The image was taken at 20X, confocal microscopy. Mean ± SEM; *p<0.05, **p<0.01, ***p<0.001 and ****p<0.0001.


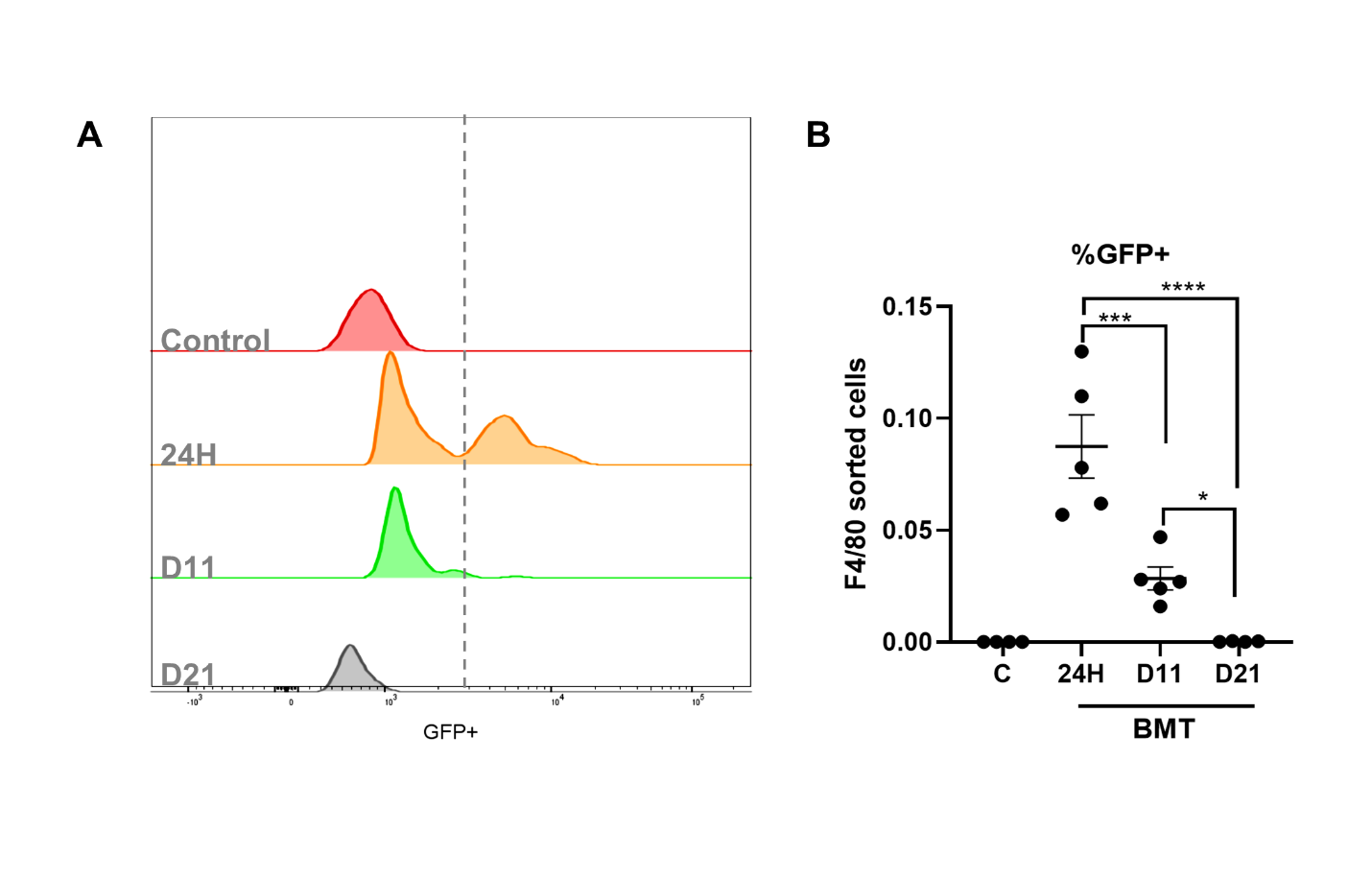


**Figure S11: GFP+ in sorted F4/80+ cells.** (A) The histogram showing the GFP+ expression at 24H as compared to control, D11 and D21 post-BMT. (B) the graph showing the percentage change in GFP+ expression of the sorted F4/80+ parent population. Mean ± SEM; *p<0.05, **p<0.01, ***p<0.001 and ****p<0.0001.


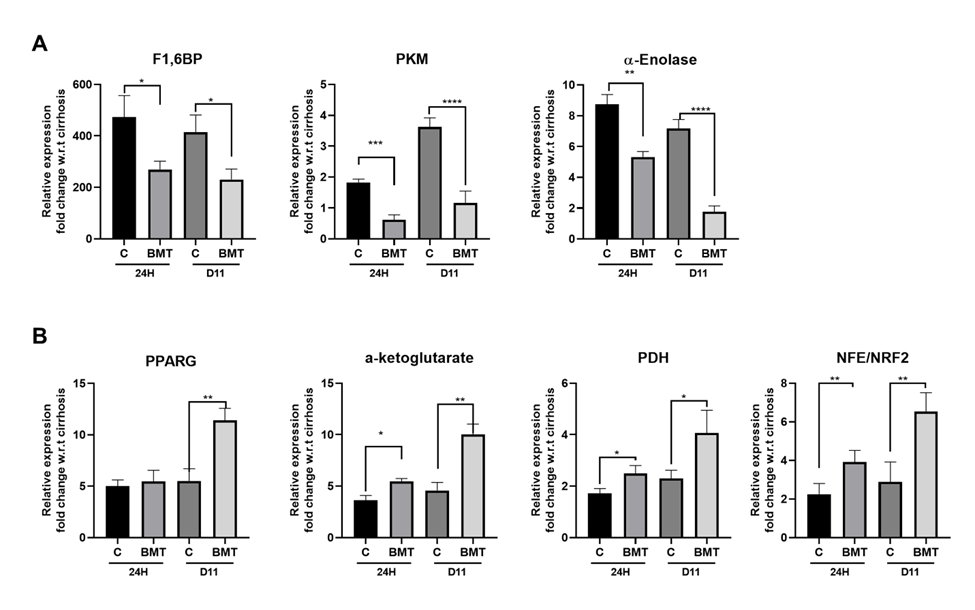


**Figure S12: RT-PCR gene expression.** (A-B) The bar charts showing the results of RT-PCR analysis, illustrating the changes in gene expression concerning cirrhosis for genes linked to glycolysis (F16BP, PKM, and α-Enolase) and (B) genes associated with OXPHOS and mitochondrial energy metabolism (PPARG, a-ketoglutarate, PDH and NRF2) at 24H and D11 in liver tissue of hBMT compared to control. Mean ± SEM; *p<0.05, **p<0.01, ***p<0.001 and ****p<0.0001.


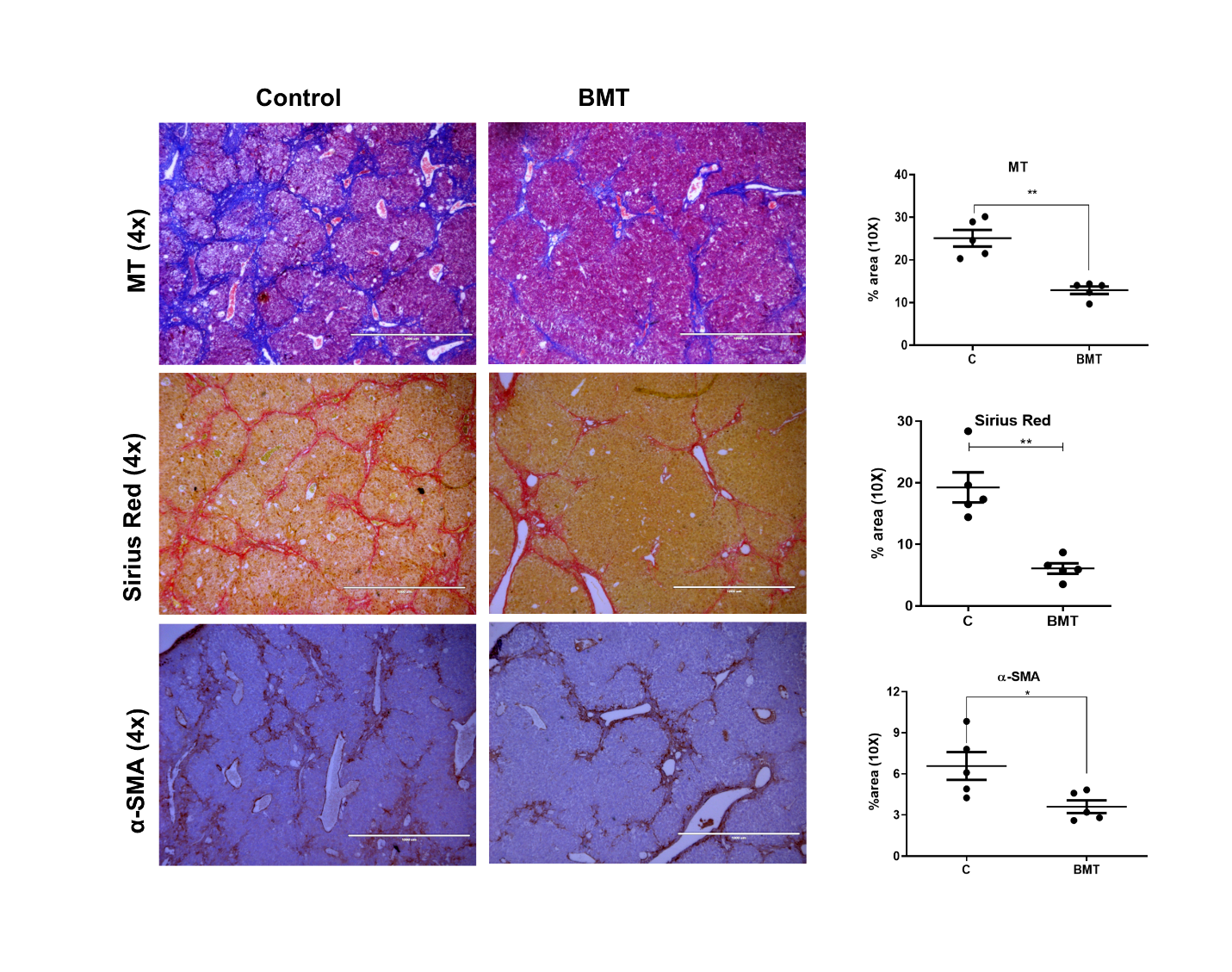


**Figure S13. Fibrosis regression post-BMT at D11.** The micrograph showing the regression of fibrosis based on MT, SR and α-SMA staining (4X), at D11 post BMT in comparison to control (N=5). The images were taken in EVOS@FL2 for different areas at 4X magnification and quantified using imageJ for different areas (10X). Mean ± SEM; *p<0.05, **p<0.01, ***p<0.001 and ****p<0.0001.


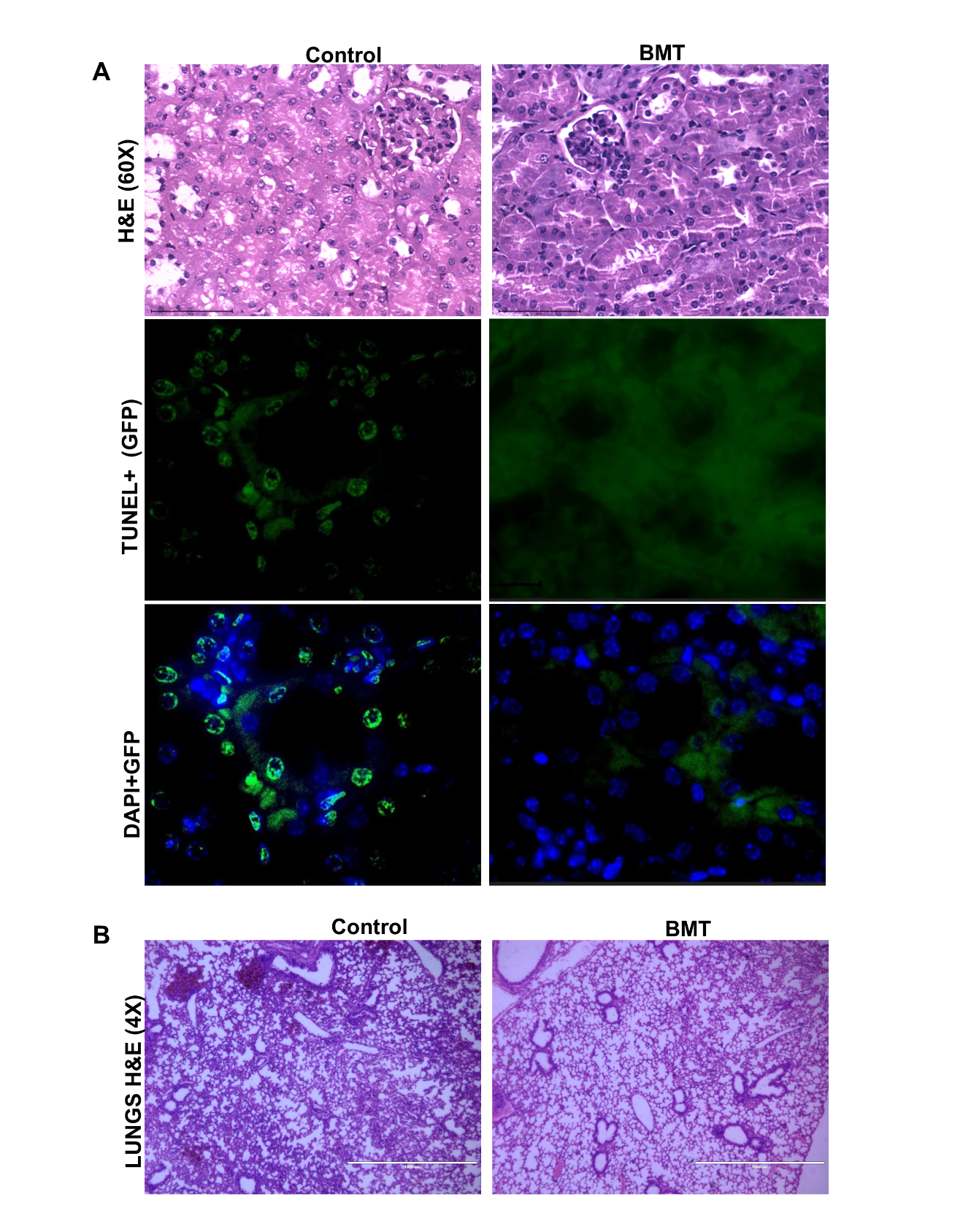


**Figure S14. Systemic organ damage.** (A) the micrographs showing the tubular necrosis (H&E and TUNEL+ staining) in controls as compared to BMT group. The TUNEL+ staining seen in control set of mice in the tubular region while BMT group showed minimum or no tubular injury. (B) The micrograph showing the pulmonary fibrosis in control set of mice as compared to BMT group. The images were taken in EVOS@FL2 for different areas at 4X, 20X and 60X magnification.

**Supplementary Table S1: Antibodies details**

***Flow cytometry Antibodies***

| **Antibodies** | **Fluorochrome** | **Manufacturer** | **Catalog** | **Dilution** |
| --- | --- | --- | --- | --- |
| Lineage (LIN) | V450 | Thermo | 88-7772-72 | 1:20 |
| C-KIT | PE-Cyanine7 | Thermo | 25-1171-82 | 1:10 |
| SCA-1 | APC | Thermo | 17-5981-82 | 1:10 |
| CD34 | APC-Cy7 | BioLegend | 128622 | 1:10 |
| Flt3 (CD135) | PE | Thermo | 12-1351-82 | 1:10 |
| F4/80 | eFlour450 | Thermo | 48-4801-80 | 1:20 |
| Nestin | PE | Invitrogen | MA5-23574 | 1:20 |
| Ter119 | APC | BioLegend | 116211 | 1:10 |
| CD45 | PE-Cy7 | BioLegend | 157205 | 1:10 |
| CD31 | APC-Cy7 | BioLegend | 102533 | 1:10 |
| Phagocytosis (E.coli beads) | FITC | Cayman | 500290l | As per the manufacturer’s protocol |

***Immunohistochemistry Antibodies***

| **Antibodies** | **Type** | **Manufacturer** | **Catalog** | **Dilution** |
| --- | --- | --- | --- | --- |
| PCNA | Polyclonal; Anti-Rabbit | Cloud-Cloud | PAA591Mi01 | 1:200 |
| Collagen I | Polyclonal; Anti-Rabbit | Thermo | PA5-29569 | 1:400 |
| cK-19 | Monoclonal; Anti-Rabbit | Abcam | ab52625 | 1:200 |
| F4/80 | Monoclonal; Anti-Rabbit | CST | 70076 | 1:200 |
| a-SMA | Monoclonal; Anti-mouse | Thermo | MA5-11547 | 1:400 |
| Nestin | Monoclonal, anti-Rabbit | Thermo | MA1-110 | 1:100 |
| TUNEL+ | FITC | Roche | 11684795910 | As per the manufacturer protocol |
| DAB EqV | Vector Laboratory | SK-4103 | 1:1 (Substrate : Chromogen) | DAB EqV |
| Secondary Antibody  (Anti-Rabbit/Rat) | ImmPRESS (Peroxidase) Polymer Detection Kit; Anti-Rabbit IgG-MP-7401 Or Anti-Rat IgG-MP-7404 | | | |
| Secondary Antibody  (Anti-Mouse) | Vector M.O.M (Mouse-On-Mouse); ImmPRESS (Peroxidase) Polymer Detection Kit; Anti-Mouse IgG; MP-2400 | | | |

**Supplementary Table S2: Reagent details**

| **Name** | **Company** | **Catalog No.** | **Lot No.** |
| --- | --- | --- | --- |
| CCL4 | Central Drug House | 56-23-5 | Product code: 490010 |
| Olive Oil | HIMEDIA | GRM6360 | 8001-25-0 |
| RPMI | HIMEDIA | RPMI-1640 | 0000622065 |
| α-MEM | Gibco | 12561-056 | 1663193 |
| GlutaMAX(100X) | Gibco | 35050-061 | 1852918 |
| Anti-Anti | Gibco | 15240-062 | 2211109 |
| FBS | HIMEDIA | RM1112 | 0000439368 |
| MEM-NEAA (Non-Essential Amino acid) (100X) | Gibco | 11140-060 |  |
| DIR | Invitrogen | D12731 |  |
| TrypLE | Gibco | 12605-010 | 1813725 |
| Ketamine | Qualket | Mfg Lic no.: G/28-B/12 | P1790 |
| Xylazine | Xylagen | Mfg Lic no.: 28/RR/AP/2009/F/R | S21308 |
| HiSep | HIMEDIA | LS001 | 0000401074 |
| GranuloSep | HIMEDIA | LS004 | 0000416641 |
| cDNA synthesis Kit | Thermo | 4368814 | -- |
| SYBER Green qPCR | GBiosciences | 786-5062 | -- |

**Supplementary Table S3: Primer’s List**

| **Primers** |  | **Sequence (5' -> 3')** | **Length** | | **Tm** |
| --- | --- | --- | --- | --- | --- |
| Fructose 1 6-bisphosphate (F16BP) | Forward | GAAACCGCCTGCAAAGGATAA | | 21 | 60.9 |
|  | Reverse | GAGGGTCTCGTGGAAAAGGAT | | 21 | 61.2 |
|  |  |  | |  |  |
| Pyruvate kinase (PKM) | Forward | AGGGGCACCCAAGTACATC | | 19 | 61 |
|  | Reverse | TGCCGGAGGAAAGTGAATGAC | | 21 | 62.4 |
|  |  |  | |  |  |
| α-Enolase | Forward | TGCGTCCACTGGCATCTAC | | 19 | 61.7 |
|  | Reverse | CAGAGCAGGCGCAATAGTTTTA | | 22 | 60.9 |
| α-Ketoglutarate (KG) | Forward | CAGCCAGTGATTCGGATTTCC | | 21 | 61 |
|  | Reverse | GCTGGTCAGATACAGCAAGTC | | 21 | 60.4 |
|  |  |  | |  |  |
| PPARG | Forward | TATGGAGTGACATAGAGTGTGCT | | 23 | 60.3 |
|  | Reverse | CCACTTCAATCCACCCAGAAAG | | 22 | 60.8 |
|  |  |  | |  |  |
| NRF2 | Forward | TAGATGACCATGAGTCGCTTGC | | 22 | 62.1 |
|  | Reverse | GCCAAACTTGCTCCATGTCC | | 20 | 61.6 |
|  |  |  | |  |  |
| Pyruvate dehydrogenase (PDH) | Forward | AGGGGCACCCAAGTACATC | | 19 | 61 |
|  | Reverse | TGCCGGAGGAAAGTGAATGAC | | 21 | 62.4 |
|  |  |  | |  |  |
| 18S | Forward | AGGGGTTCGGGATTTGTGC | | 19 | 60.3 |
|  | Reverse | GACAAAACCCGCCGCGA | | 17 | 64.7 |
